# Partial depletion of circulating neutrophil granulocytes in mice exacerbates the inflammatory response and hypothermia during LPS induced severe systemic inflammation

**DOI:** 10.3389/fimmu.2025.1578590

**Published:** 2025-06-04

**Authors:** Jessica Hernandez, Fabian Johannes Pflieger, Julia Schäffer, Leona Bähr, Jenny Schneiders, Thomas Reichel, Marita Meurer, Benjamin Lamp, Natali Bettina Bauer, Karsten Krüger, Lois Harden, Maren von Köckritz-Blickwede, Christoph Rummel

**Affiliations:** ^1^ Institute of Veterinary Physiology and Biochemistry, Faculty of Veterinary Medicine, Justus Liebig University Giessen, Giessen, Germany; ^2^ Institute of Sports Science, Department of Exercise Physiology and Sports Therapy, Justus Liebig University Giessen, Giessen, Germany; ^3^ Institute for Biochemistry, University of Veterinary Medicine Hannover, Hannover, Germany; ^4^ Research Center for Emerging Infections and Zoonoses, University of Veterinary Medicine Hannover, Hannover, Germany; ^5^ Institute of Virology, Faculty of Veterinary Medicine, Justus Liebig University Giessen, Giessen, Germany; ^6^ Clinic for Small Animals (Internal Medicine, Clinical Pathophysiology, and Clinical Pathology), Justus Liebig University Giessen, Giessen, Germany; ^7^ Brain Function Research Group, Department of Physiology, School of Biomedical Sciences, Faculty of Health Sciences, University of the Witwatersrand, Johannesburg, South Africa; ^8^ Center for Mind Brain and Behavior (CMMB), Universities Giessen and Marburg, Marburg, Germany; ^9^ Translational Neuroscience Network Giessen (TNNG), Justus Liebig University, Giessen, Germany

**Keywords:** neutrophil granulocytes, neutropenia, inflammation, hypothermia, sickness response, immune-to-brain signaling

## Abstract

**Introduction:**

During acute inflammation, immune-to-brain signaling plays a pivotal role in the generation of sickness responses such as fever or hypothermia. Neutrophil granulocytes (NG) are a crucial component of the immune system and modulate inflammation. Moreover, neutropenic fever is a severe condition for immunocompromised patients that can be life threatening. Using a mouse model of partial NG depletion, we aimed to investigate how neutropenia alters immune-to-brain signaling and the development of sickness responses during high-dose-LPS-induced inflammation.

**Methods:**

To deplete NGs, mice were injected intraperitoneally (IP) with heterologous anti-polymorphonuclear leukocyte serum at 1:4 ratio in PBS (PMN, 1.82 mg/kg IgG) or normal rabbit serum (NRS, 1 mg/kg IgG) as a control. To induce inflammation, mice were injected IP with lipopolysaccharide (LPS, 2.5 mg/kg) or PBS as a control 24 h after PMN or NRS. Physiological parameters were documented using a telemetric system that continuously recorded: food and water intake, locomotor activity, and core body temperature. At 4 h or 24 h after LPS-stimulation, brain and serum samples were collected and analyzed for peripheral and brain inflammatory markers.

**Results:**

After stimulation with LPS, PMN-pretreated mice showed neutropenia (significantly by ~25% of the control value) and attenuated NG recruitment to the brain in a structure dependent manner. LPS-induced hypothermia was more severe in PMN-pretreated mice while other physiological parameters were only altered by LPS alone. Additional analyses in NG depleted mice revealed that corticosterone levels showed an early reduced but late increased magnitude, and circulating cytokines like interleukin-10 were exacerbated during LPS-induced inflammation. Despite a weak overall impact on the brain, the hypothalamus of neutropenic mice presented exacerbated LPS-induced levels of IL-6, a key mediator of inflammation, compared to immunocompetent control mice.

**Discussion:**

Overall, we found that partial NG depletion exaggerates the peripheral inflammatory response and this strong peripheral reaction may contribute to the exacerbation of sickness symptoms most likely involving circulating IL-10 with strong implications for clinical cases of neutropenic patients.

## 1 Introduction

Sepsis is a serious condition accompanied by an extreme systemic inflammatory response to an infection and is one of the leading causes of mortality in the intensive care unit (1, 2). The host response to sepsis includes the development of both, pro-inflammatory responses and anti-inflammatory immune suppression (3, 4). Patients who have undergone myelosuppressive chemotherapy treatments are susceptible to infections and have a high risk of developing sepsis (3, 5). Additionally, these patients have also been observed to develop febrile neutropenia, an intense and persistent fever. Current treatments for sepsis rely heavily on infection management, aggressive fluid administration, and organ support to increase survival rates. Despite advances in the treatment of sepsis, survivors often have severe long-term impairments (2, 6–8).

The exact origin of fever associated with neutropenia remains unknown in part due to the poorly understood functions of neutrophil granulocytes (NG) (3, 5, 9). However, neutropenic fever, characterized by an elevated body temperature in individuals with low NG counts (9, 10), is a significant clinical concern and is associated with increased mortality during septic inflammation (11). Since reduced NGs compromise the body’s ability to mount an appropriate immune response, neutropenic fever could signal a potentially life-threatening infection and is often considered a medical emergency (11, 12). Even today, it is challenging to treat neutropenic fever and patients still require careful monitoring (12, 13). Understanding the mechanisms underlying neutropenic fever is crucial for developing effective therapeutic strategies.

As the most abundant cell type of the innate immune system in humans and rodents, NGs are of particular importance during sepsis (14). The dysregulation of NGs can lead to their accumulation in the circulation, impaired bacterial clearance, and increased production of interleukin (IL)-10 (15). The increase in IL-10 can subsequently exacerbate disease progression by promoting the development of lymphopenia and the reduction of T-cell propagation (15, 16). Additionally, the release of neutrophil extracellular traps (NETs) by NGs, which normally serve to capture and clear pathogens, is elevated in septic patients and promote a hypercoagulable state increasing the risk for thrombosis and hemorrhage (17–21). However, in contrast to these pro-inflammatory actions, the anti-inflammatory properties of NGs have also been documented. Indeed, studies have previously shown that NGs can release the anti-inflammatory immune-mediator IL-1 receptor antagonist (human) and inducible nitric oxide synthase (mouse) in response to lipopolysaccharide (LPS)-stimulation *in vitro* and during a model of ischemic brain injury (22, 23). The paradoxical pro- and anti-inflammatory nature of NGs highlights the importance of increasing our understanding of the role of NGs during sepsis. For example, the capacity of NGs to exert dual functions may contribute to the resolution of the sickness response during infection or inflammation.

Over the course of septic inflammation, various brain pathologies and brain-mediated sickness responses, including lethargy, anorexia, and fever/hypothermia can occur (24–26). Indeed, encephalopathy can develop during sepsis and it is a severe neuroinflammatory condition that impacts cognitive functions, causes altered mental states, and is associated with increased mortality (27–31). It is well established that immune-to-brain communication transmits information from the periphery to the brain and plays an important role in brain functions in addition to regulating the immune response (32, 33). In particular, interactions at brain structures, which lack a tight blood-brain barrier, such as the circumventricular organs (CVOs), are able to detect cytokine signals and transmit information to the brain (34–36). While many studies have focused on the humoral route of immune-to-brain communication to transmit the majority of information, there is also evidence of peripheral immune cells infiltrating the brain and influencing the inflammatory process including modulating brain functions (28, 32, 33, 37). During systemic inflammation, both monocytes and NGs have been observed in the brain. In a mouse model of cerebral ischemia induced by LPS administration, brain pathology associated with inflammation has been shown to be exacerbated via NG-dependent mechanisms (38–40). We have previously shown, using a mouse model of severe systemic inflammation, that NG recruitment to the brain occurs in a time-dependent and region-specific manner (41). Moreover, Aguilar-Valles and colleagues (2014) demonstrated that NG depletion inhibited depression-like behaviors in surviving mice during septic-like inflammation (28). Despite the existing evidence of NG infiltration into the brain and impacts on brain-controlled sickness responses during severe systemic inflammation, little is known about the effects of NGs on the central nervous system (28, 41, 42).

Thus, in the present study, we tested the hypothesis that NGs play a protective role by modulating circulating cytokines, such as IL-10, as well as participating in immune-to-brain communication and contributing to the brain-controlled sickness responses associated with severe systemic inflammation. We tested our hypothesis in a mouse model of sepsis using antibody-targeted depletion of NGs followed by an intraperitoneal (IP) injection of a high LPS dose and examined markers of peripheral and brain inflammation. To assess sickness responses, locomotor activity and core body temperature (Tb) were continuously recorded and food and water intake were monitored over the course of the experiment using telemetric systems. Improving our understanding of the role played by NGs during severe systemic inflammation, may lead to innovative therapeutic options and improved quality of life for patients with chemotherapy-induced neutropenia or febrile neutropenia.

## 2 Materials and methods

### 2.1 Animals

All mice were obtained through in-house breeding in groups and maintained under specific pathogen-free conditions on a 12 h light/dark cycle at 22°C ± 1°C and 50% ± 5% humidity with food and water available *ad libitum*. Original C57BL/6J breeding pairs were obtained from Charles River Laboratories (Sulzfeld, Germany). Male mice aged 6–8 weeks, weighing approximately 25–30 g, were used for experiments. Intra-abdominal radio transmitters to record Tb and locomotor activity (TA-F10, Data Sciences International, St. Paul, MN, USA) were surgically implanted about one week prior to the start of the experiments. Surgical implantation of the transmitters was performed using an antagonizable IP anesthetic composed of fentanyl (10 ml/kg; Dechra Veterinary Products, Aulendorf, Germany), midazolam (10 ml/kg; Henry Schein Dental, Hamburg, Germany), and medetomidine (10 ml/kg; Henry Schein Dental, Hamburg, Germany). An antagonist comprised of atipamezol (10 ml/kg; Henry Schein Dental, Hamburg, Germany) and flumazenil (10 ml/kg; Henry Schein Dental, Hamburg, Germany) was administered subcutaneously to reverse the anesthesia. Meloxicam (1 mg/kg body weight; Boehringer Ingelheim, Ingelheim, Germany) was administered orally for surgical analgesic treatment. Experimental cages were positioned on receiver plates (DSI PhysioTel™ RPC-1, Data Sciences International, St. Paul, MN, USA) and Ponemah^®^ P3P software was used to continuously record Tb and locomotor activity (Data Sciences International, St. Paul, MN, USA). Food and water were located on scales outside the cage to continuously record consumption. Mice had access to the food and water by a tunnel or rodent sipper tip. The animal experiments were approved by local authorities (Regierungspräsidium Giessen: GI 18/2 Nr. G 72/2017) and performed in accordance with the German Animal Welfare Act and international legislation.

### 2.2 Treatment and experimental protocol

About four to five days after surgery, mice were switched from group housing in conventional cages to individual housing in experimental cages where they continued to recover for an additional three to five days. Once individually housed in experimental cages, food and water intake was recorded daily. Two days prior to the start of the experiment, recordings for Tb and locomotor activity were recorded to establish baseline values. To deplete NGs in the circulation, an IP injection of heterologous anti-polymorphonuclear leukocyte serum (PMN, at a dose of 5 ml/kg corresponding to 1.82 mg/kg total IgG; No.: WAK-AIA31140 supplied by WAK-Chemie Medical GmbH, Germany with the original US catalog number AIA31140, lot: 6326, Accurate Chemical and Scientific, Westbury, NY, USA) diluted in sterile pyrogen free 0.9% phosphate buffered saline (PBS; PAA, Pasching, Austria) was administered according to a well-established model (28, 43–47). Controls were injected with an identical dilution and equal volume of normal rabbit serum (NRS; at a dose of 5 ml/kg corresponding to 1.0 mg/kg total IgG, No.: WAK-AIS403, lot: 0925, Accurate Chemical and Scientific, Westbury, NY, USA). To induce severe systemic inflammation, mice were injected IP with LPS (2.5 mg/kg; derived from Escherichia coli, serotype 0111:B4, lot: 078M4039V, Sigma-Aldrich, Munich, Germany), diluted in PBS, 24 h after the initial injection with PMN or NRS. Indeed, we have previously shown that this LPS-dose induces severe systemic inflammation in mice (48–50). Controls were injected with an equal volume of PBS.

A dose-finding trial identified the anti-serum amount that effectively reduced NGs. To find an effective dilution of PMN that sufficiently depleted NGs without causing lethality, three different IgG doses diluted in PBS were tested: 1:4 dilution ratio at 1.82 mg/kg, 1:1.5 dilution ratio 3.64 mg/kg, and 9.1 mg/kg (undiluted). Inoculation with LPS caused an increase in circulating levels of NGs and recruitment of NGs to the brain, effects that were significantly attenuated (~20-25%) by pre-treatment with PMN at 1.82 mg/kg IgG and 3.64 mg/kg IgG dilutions (**Supplementary Figures 1, 2**). The undiluted dose of PMN proved to be too severe and was lethal in combination with LPS and thus not tested further. The only dose of PMN that did not cause any fatalities during these preliminary experiments was the 1.82 mg/kg IgG dilution and therefore, selected for the experiments. However, over the course of our experiments, two mice pre-treated with PMN (1.82 mg/kg IgG dilution) died as a result of LPS-induced inflammation.

All injections were administered at a total injection volume of 5 ml/kg between 9:00 am and 11:30 am. At 4 h or 24 h post inoculation (p.i.) with LPS or PBS, the mice were killed during terminal anesthesia induced with an IP injection of pentobarbital (160 mg/kg, Boehringer Ingelheim, Ingelheim, Germany). Thereafter, the mice were transcardially perfused with ice-cold 0.9% saline. A sterile heparinized syringe was used to collect blood samples via cardiac puncture prior to the perfusion and afterwards, brains were quickly removed and frozen on powdered dry ice. All samples were stored at -80°C until analyses were completed.

### 2.3 Determination of total IgG from NRS and PMN serum in a sandwich ELISA

The samples were pre-diluted 1:20 in PBS with Tween (PBSt) before starting the test procedure. This pre-diluted stock was applied to generate a 10-fold dilution row in PBSt ranging from 1:20 to 1:20^8^. goat-anti-rabbit IgG (SBA-4050-01; Biozol, Hamburg, Germany) was dissolved in ELISA coating buffer (0.1 M sodium carbonate, pH 9.5) and diluted to a final concentration of 10 µg/ml. ELISA 96-well plates (Nunc MaxiSorp; Life Technologies, Darmstadt, Germany) were coated with 100 µl of capture antibody overnight and blocked using a commercial blocking reagent (ROTI-Block; Carl Roth, Karlsruhe, Germany) for 1 h at room temperature (RT). The microwells were washed twice with approximately 300 μL PBSt per well with thorough aspiration of microwell contents between washes. The buffer was allowed to sit in the wells for about 20 seconds before aspiration. Then, the diluted samples, blanks, and a rabbit IgG-standard dilution row (ranging from 50 µg/ml to 5 pg/ml) were pipetted in the microwells in triplicates. After an incubation period of 1 h at RT, the microwells were washed as described above. After an additional wash step, the wells were emptied and tapped on absorbent paper towel to remove excess PBSt. 50 μL of diluted HRP-goat-anti-rabbit conjugate was added to all wells (1 mg/ml diluted 1:5,000 in PBSt). The microwells were covered with an adhesive film and incubated at RT for 1 h. Then, the microwells were washed 4 times with PBSt and 1 time with PBS. Immediately after washing, 100 μL of TMB substrate solution was pipetted to all wells. The microwell plate was incubated at RT for 15 min, before the substrate reaction was stopped by quickly pipetting 100 μL of ELISA-stop solution (1N sulfuric acid) into each well. Results were read immediately after the stop solution was added using an ELISA-reader at 450 nm as the primary wave length and 620 nm as the reference wavelength (Spark; Tecan, Männedorf, Schweiz).

### 2.4 Tissue processing

Coronal 20 μm brain sections of multiple brain structures including the vascular organ of the lamina terminalis (OVLT, bregma 0.62-0.38 mm), the median preoptic nucleus (MnPO, bregma 0.62-0.14 mm), the subfornical organ (SFO, bregma -0.1- -0.82 mm), and the paraventricular nucleus (PVN, bregma -0.58- -1.22 mm) were cut using a cryostat (CryoStar NX50; Thermo Fisher Scientific, Dreieich, Germany). Sections were thaw-mounted on poly-L-lysine coated glass slides and stored at -80°C for immunohistochemistry. Additional sections (bregma 0.38 mm- -1.50mm) were stacked on glass slides so the hypothalamus could be dissected, divided in two (left and right hemisphere), and stored at -80°C for RNA-extraction or protein isolation.

### 2.5 Leukocyte analysis

#### 2.5.1 Hematological analysis

Complete leukocyte analysis was performed on whole blood samples from preliminary experiments, immediately following the perfusion, using an ADVIA 2120 automated hematology analyzer with the veterinary software version 5.3.1-MS and the mouse setting (Siemens Healthcare, Erlangen, Germany). Hemoglobin concentrations and red blood cell volume were determined using an established combination method of cyanide-free photometric measurements and flow cytometry (51). For white blood cell and differential count, the analyzer used laser light scatter at a wavelength of 670 nm, cytochemical myeloperoxidase staining, and differential white blood cell lysis in two separate channels, the peroxidase and baso/lobularity channel. Based on cell size, myeloperoxidase staining intensity, and nuclear lobularity, a six-part leukocyte differential count was obtained including NGs, lymphocytes, monocytes, eosinophils, basophils, and large myeloperoxidase-negative cells (large unstained cells, LUC) such as reactive lymphocytes or plasma cells (data not shown). Internal quality controls were performed daily using three concentrations of quality control materials. Annual calibrations of the hematology instrument were carried out by the manufacturer. Hematological analysis confirmed that PMN significantly reduced the amount of circulating NG (**Supplementary Figures 1, 2**).

During the experiments, total leukocyte populations were determined for each animal using the Leuko-Tic^®^ kit (Bioanalytic, Umkirch, Germany) according to the manufacturer’s instructions.

#### 2.5.2 Peripheral blood smears

Blood smears were analyzed for leukocyte differential count using a May-Gruenwald-Giemsa staining technique. Samples were incubated in 100% May-Gruenwald (Merck, Darmstadt, Germany) solution for 3 min, washed in double distilled water for 1 min, incubated in 12% Giemsa solution (Merck, Darmstadt, Germany) for 15 min, washed in double distilled water for 1 min, and allowed to air dry. All blood smears were protected from light and stored at RT.

### 2.6 Magnetic luminex assay

#### 2.6.1 Serum

A custom-made mouse magnetic bead-based assay obtained from Bio-Techne (Bio-Techne, Abingdon, Oxon, UK) was used to analyze serum levels of the following selected inflammatory mediators: granulocyte colony-stimulating factor (G-CSF), CCL5 (RANTES), CXCL1, CXCL2, tumor necrosis factor (TNF)α, IL-10, and IL-6. Serum samples were diluted at a 1:1 dilution ratio and measurements were performed according to the manufacturer’s instructions. The assay was performed with the Luminex MAGPIX^®^ system (Luminex, Austin, TX, USA).

#### 2.6.2 Hypothalamus

Tissue samples from the hypothalamus (~18.4 mg per sample) were homogenized in 300 μl of sonication buffer (100mM amino-n-caproic acid, 10mM EDTA, 5mM benzamidine, and 200μM phenylmethyl sulfonyl fluoride) in Tris buffered saline (Carl Roth, Karlsruhe, Germany). All reagents were purchased from Sigma-Aldrich unless otherwise stated. Samples were sonicated for 20 sec followed by a centrifugation step at 13,200 rpm at 4°C for 10 min. Supernatants were collected and total protein levels per sample were determined using a BCA protein assay (Thermo Fisher Scientific, Waltham, MA, USA). The following selected inflammatory mediators: CCL2, CCL5, CXCL1, CXCL2, CXCL5, granulocyte-macrophage colony-stimulating factor (GM-CSF), interferon (IFN)γ, IL-1β, IL-2, IL-6, IL-10, IL-17, and TNFα were analyzed via custom-made mouse magnetic bead-based assays from Bio-Techne (Bio-Techne, Abingdon, Oxon, UK) or Thermo Fisher (CXCL2, CXCL5; Thermo Fisher Scientific, Waltham, MA, USA). Samples were diluted at a 1:1 dilution ratio and measurements were performed according to the manufacturer’s instructions. The assay was performed with the Luminex™ 200™ system (Thermo Fisher Scientific, Waltham, MA, USA) and analyzed with xPONENT^®^ Software from Thermo Fisher Scientific (Waltham, MA, USA).

### 2.7 Corticosterone ELISA

Corticosterone levels in serum were measured using a specific mouse ELISA (Arbor Assays; Arbor Assays, Ann Arbor, MI, USA) according to the manufacturer’s instructions with a minimum detection limit of 18.6 pg/ml.

### 2.8 Immunohistochemistry

For the NG marker myeloperoxidase (MPO) and signal transducer and activator of transcription 3 (STAT3) staining, frozen brain sections were air-dried for 10 min (5 min for STAT3) and fixed in 2% paraformaldehyde (Sigma-Aldrich, Munich, Germany) diluted in PBS for 10 min. After washing three times with PBS, the sections were incubated at RT for 1 h using blocking solution containing 10% normal donkey serum (NDS; Biozol, Eching, Germany) and 0.3% (MPO) or 0.1% (STAT3) Triton X-100 (Sigma-Aldrich, Munich, Germany) in PBS. The immunofluorescent primary antibodies used included: rabbit anti-MPO (dilution 1:600; A0398, Dako, Glostrup, Denmark), rabbit anti-mouse STAT3 (dilution 1:2000; sc-482, Santa Cruz Biotechnology, Dallas, TX, USA), and sheep anti-von Willebrand factor (vWF, dilution 1:2000; SARTW-IG, Affinity Biologicals, Ancaster, Canada). Primary antibodies were diluted in the blocking solution and sections were incubated overnight at 4°C. After washing three times, the sections were incubated with the secondary antibodies diluted in the blocking solution for 2 h at RT. The secondary antibodies used included: Alexa 488-conjugated donkey anti-sheep IgG (dilution 1:500; A11015, Life Technologies, Carlsbad, CA, USA) and Cy3-conjugated donkey anti-rabbit IgG (dilution 1:600; 711-165-152, Jackson Immuno Research Europe, Newmarket, UK). After additional three washes with PBS, the cell nuclei were stained with 4.6-diamidino-2-phenylindole (DAPI, dilution 1:5000 in PBS, MoBiTec, Göttingen, Germany) for 10 min followed by a final set of three washes with PBS. Once the staining was completed, sections were cover slipped using Citifluor (Citifluor, London, UK) and stored at 4°C until they could be imaged. MPO images were collected within three days but images for STAT3 were collected the same day.

#### 2.8.1 Assessment of neutrophil granulocyte recruitment

Images were acquired using a light/fluorescent Olympus BX50 microscope (Olympus Optical, Hamburg, Germany; x40 objective lens, UPlanFI; numeric aperture, NA ¼ 0.75; x20 objective lens, UPlanFI; numeric aperture, NA 0.50) with a black and white Spot Insight camera (Diagnostic Instruments, Visitron Systems, Puchheim, Germany). Microphotographs were taken consecutively for each staining with the same exposure times using MetaMorph 7.7.5.0 software (Molecular Devices, Downingtown, PA, USA). Individual images were combined with RGB color images using MetaMorph 5.05 software and optimized for contrast and brightness (all images were processed the same way) using Adobe Photoshop 6.0 (Adobe Systems Incorporated, San Jose, CA, USA).

For preliminary experiments assessing the effects of different PMN doses, stained with MPO and vWF, images were taken of the OVLT, SFO, Plexus, and PVN and a semi quantitative evaluation using a 5-level scale (-, no signals detectable; ±, single signal in some cases; +, low density; ++, moderate density; +++, high density of signals) was used to evaluate MPO expression. At each structure, 1–3 mice with 1–12 sections per mouse were analyzed and subsequently averaged for each animal. The overall effect of pre-treatment with PMN vs. NRS on NG recruitment to the brain was evaluated by pooling the means of each group regardless of brain structure.

NG recruitment to the SFO and PVN was assessed by counting MPO^+^ cells at each structure. Between 4–8 mice with 1–4 sections per mouse were analyzed and subsequently averaged for each animal.

#### 2.8.2 Assessment of STAT3 immunofluorescence

Images were acquired using a Leica THUNDER Imager 3D Tissue System with a K5 Microscope camera (x40 objective lens, HC PL APO; numeric aperture 0.95 CORR). Microphotographs were taken consecutively for each staining with the same exposure times. Individual images went through Computational Clearing using THUNDER Imaging and optimized for contrast and brightness (all images were processed the same way) using Adobe Photoshop 6.0 (Adobe Systems Incorporated). All systems used were obtained from Leica Microsystems in Wetzlar, Germany unless otherwise stated.

Images were taken of the OVLT at the 4 h time point and a semi quantitative evaluation, using the same 5-level scale as previously mentioned, was used to evaluate STAT3 immunoreactivity. For each group, 3 mice with 3–5 sections per mouse were analyzed and subsequently averaged for each animal.

#### 2.8.3 NET staining and assessment

For staining the NET markers, DNA/histone complex (DNA/His) and citrullinated histone H3 (H3Cit), frozen brain sections were shortly fixed in 2% paraformaldehyde (Sigma-Aldrich, Munich, Germany). After washing three times with PBS and blocking 1 h in blocking buffer (1% BSA, 5% goat serum, 2% cold water fish gelatine, 0.05% Tween 20 and 0.05% Triton X100 in TBS) samples were stained overnight with mouse monoclonal anti DNA/Histone (Millipore MAB3864, 2.2 mg/ml, 1:300) and rabbit anti-H3Cit (ABCAM ab5103, 1 mg/ml; 1:50). Respective isotype staining was performed with IgG2a from murine myeloma (Sigma M5409, 0.2 mg/ml; 1:27) and rabbit IgG, whole molecule (Sigma I5006, 1,16 mg/ml; 1:58) in blocking buffer. After washing three times with PBS, second antibody staining was performed with goat anti-mouse IgG Alexa fluor plus 488 (Invitrogen A32723, 2 mg/ml; 1:500) and goat anti-rabbit IgG Alexa fluor 633 (Thermo Scientific, A21070, 2 mg/ml) 1:500). DNA counterstaining was done after three washing steps in PBS by mounting the samples with Dapi containing Prolong Gold (Molecular Probes, P36931). Images were taken with a Leica TCS SP5 AOBS confocal inverted-base fluorescence microscope with HCX PL APO 40× 0.75–1.25 and HCX PL APO lambda blue 63× 1.40 oil immersion objectives. The settings were adjusted according to the isotype controls.

### 2.9 Quantitative RT-qPCR

Total RNA was extracted from hypothalamic tissue samples (~18.4 mg per sample) using Trizol (Thermo Fisher, Waltham, MA, USA) according to the manufacturer’s instructions. Quality of RNA showed a purity ratio OD260/280 between 1.9-2.1. Reverse transcription of 1 μg total RNA was carried out using 50 U murine leukemia virus (MULV) reverse transcriptase, 10 mM dNTP mix, and 50 μM random hexamer (Sigma-Aldrich, Munich, Germany) in a 20 μl reaction volume. Afterwards, reverse transcription real-time PCR (RT-qPCR) was performed in duplicate using a preoptimized primer/probe mixture and TaqMan Gene Expression Master Mix (Thermo Fisher, Waltham, MA, USA). Based on a previous assessment of 12 commonly used housekeeping genes (48), GAPDH (4352339E-1009032; Applied Biosystems, Waltham, MA, USA) was selected as the best and was used to normalize quantities of cDNA. Values were calculated as an x-fold difference in expression from the control sample determined as 1 (NRS+PBS 4 h or 24 h) using the ΔΔC_T_-method. Assay IDs for the analyzed genes are as follows: CD68 (Mm03047340_m1), CD163 (Mm00474091_m1), COX-2 (Mm00478374_m1), CXCL1 (Mm04207460_m1), ELANE (Mm00469310_m1), IL-6 (Mm00446190_m1), IL-10 (Mm00439614_m1), mPGES (Mm00452105_m1), NF-IL6 (Mm00843434_s1), NFκBiα (Mm00477798_m1), SOCS3 (Mm00545913_s1), and TNFα (Mm00443258_m1). All primers were purchased from Thermo Fisher Scientific unless otherwise stated.

### 2.10 Data analysis

Telemetry data for Tb and locomotor activity were analyzed with IBM SPSS Statistics 26 or 29 software (IBM Corporation, Armonk, New York, USA) using two-way repeated measures analysis of variance (ANOVA; factors: condition [NRS or PMN] and treatment [PBS or LPS] over time). Data were divided into 1 h-intervals (1 h-4 h p.i.) or 2 h-intervals (4 h-24 h p.i.) for analysis and a Holm-Bonferroni *post-hoc* test was performed to correct for multiple testing and to evaluate main effects and interactions. Analysis of baseline measurements from -48 h- -24 h p.i. and -24 h-0 h p.i. were also analyzed in 2 h-intervals using the same method. When Tb was inconsistent with those previously observed within the same treatment groups and the inflammatory cytokine profile in the serum was also absent, indicating a failure of the LPS-injection, mice were excluded from analysis. In total four mice were excluded: 2 PMN+LPS (24 h), 1 PMN+LPS (4 h), and 1 NRS+LPS (24 h). Activity counts/min > 29 most likely represent artifacts and were also excluded and replaced with an average of the activity counts directly preceding and after it.

Preliminary data for circulating and brain levels of NGs were analyzed by unpaired t-test (GraphPad Prism 5 and 9 Software, San Diego, CA, USA). All other data were analyzed separately at either 4 h or 24 h p.i. using a two-way ANOVA (factors: condition [NRS or PMN] and treatment [PBS or LPS]) followed by a Tukey *post-hoc* test (GraphPad Prism 7 and 9 Software, San Diego, CA, USA). Only for the NET markers H3Cit and DNA/His we included an additional analysis with the factors treatment and time. Analyses using the ROUT method were applied to identify and exclude outliers (52), in these instances, exclusions are specified in **Supplementary Table 1**.

## 3 Results

### 3.1 Circulating lymphocyte and monocyte populations were not altered by neutrophil granulocyte depletion

Further analyses of leukocyte populations in circulation confirmed that at 4 h p.i. PMN was efficient in reducing plasma NGs without significantly altering other cellular populations (**Supplementary Figure 3**). At 4 h p.i. leukocyte populations, overall, were reduced in an LPS-dependent (*p* < 0.001) and PMN-dependent (*p* < 0.01) manner (**Supplementary Figure 3A**). In assessing populations of NGs, lymphocytes, and monocytes at 4 h p.i., we found that LPS did not affect NGs but decreased lymphocyte (*p* < 0.001) and increased monocyte (*p* < 0.001) populations regardless of NRS or PMN pre-treatment (**Supplementary Figures 3B–D**). Overall, pre-treatment with PMN was able to reduce circulating NG counts at 4 h p.i. (*p* < 0.001). While lymphocytes were unaffected by PMN pre-treatment, a PMN effect on monocytes was detected that indicated a minor increase in percentage (*p* < 0.01). Raw cell counts of NGs, lymphocytes, and monocytes are shown as descriptive data (**Supplementary Figures 3E-H**).

By 24 h p.i. the LPS-dependent (*p* < 0.01) and PMN-dependent (*p* < 0.05) reductions in leukocytes were still present, indicating that both treatments may have a prolonged effect to decrease cell levels (**Supplementary Figures 3I-L**). In assessing populations of NGs, lymphocytes, and monocytes at 24 h p.i., we found that LPS significantly increased NGs (*p* < 0.001) and monocytes (*p* < 0.001) but decreased lymphocyte (*p* < 0.001) populations regardless of NRS or PMN pre-treatment (**Supplementary Figures 3J-L**). Pre-treatment with PMN continued to have an effect on NG levels (*p* < 0.01) indicating an overall reduction. Lymphocytes remained unaffected by PMN but the minor increase in monocyte percentages for PMN pre-treated mice compared to the NRS pre-treated controls was still detectable (*p* < 0.05). Raw cell counts of NGs, lymphocytes, and monocytes were shown as descriptive data (**Supplementary Figures 3M-P**).

### 3.2 Hypothermia was exacerbated in neutrophil granulocyte depleted mice after high-dose-LPS-stimulation

**Figure 1:**
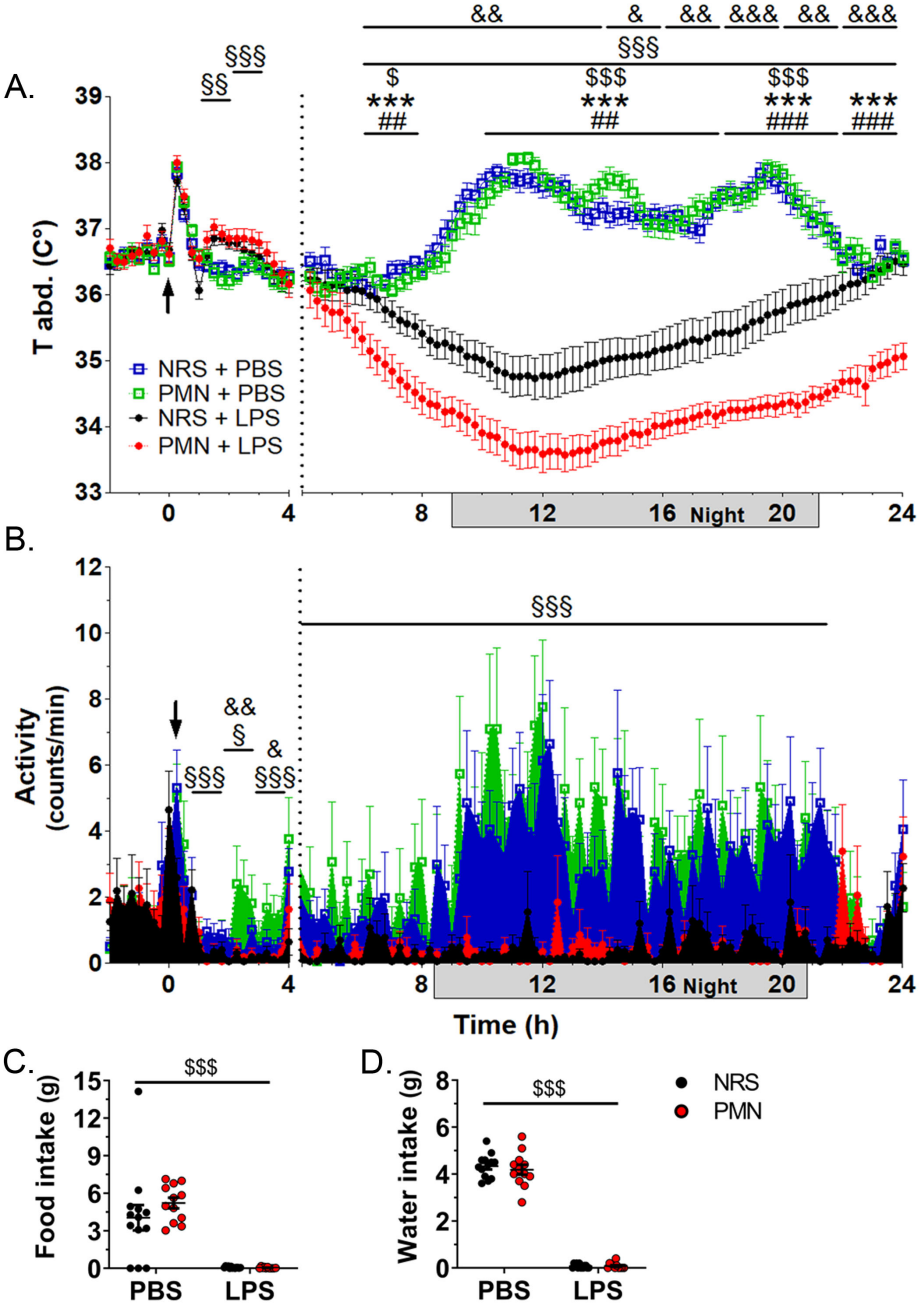
Neutrophil granulocyte depletion exacerbated LPS-induced hypothermia but not other physiological parameters. An intra-abdominal transmitter continuously recorded the physiological parameters of mice pre-treated with anti-polymorphonuclear serum (PMN) or normal rabbit serum (NRS) by intraperitoneal (IP) injection and subsequently challenged with IP lipopolysaccharide (LPS, 2.5 mg/kg) or phosphate buffered saline (PBS) as indicated by an arrow. **(A)** Core body temperature (T abd.). **(B)** Activity counts per minute. Data are presented as line graphs over time, grey boxes indicate the dark or night cycle, with mean ± SEM (n=23–24 [-2–4 h p.i.] / n=14 [4–24 h p.i.]). **(C)** Total food intake at 24 h p.i. with LPS. **(D)** Total water intake at 24 h p.i. with LPS. Data are presented as dotplots with mean ± SEM (n=11-13). **(A, B)** Statistical analysis were performed by Two-way repeated measures ANOVA and a Holm-Bonferroni *post-hoc* test with the effects: ^&^PMN, ^§^LPS; ^$^NRS+PBS vs. NRS+LPS, *PMN+PBS vs. PMN+LPS, ^#^NRS+LPS vs. PMN+LPS. **(C, D)** Statistical analysis were performed by Two-way ANOVA with the main effect ^$^LPS (^§^ *p*<0.05, ^§§^ *p*<0.01, ^§§§^ *p*<0.001, ^&^ *p*<0.05, ^&&^ *p*<0.01, ^&&&^ *p*<0.001, ^$^ *p*<0.05, ^$$$^ *p*<0.001, ^##^ *p*<0.01, ^###^ *p*<0.001, ***p<0.001).

**Figure 1** shows the development of sickness responses in mice after 2.5 mg/kg LPS. The Tb of both, NRS and PMN pre-treated control mice, showed a typical circadian day-night rhythm as well as a short-lasting peak due to the stress of handling and the injection after PBS, while groups treated with LPS became hypothermic (**Figure 1A**). Immediately following treatment, both NRS and PMN groups developed a short-lasting fever (main effect of LPS) for approximately 2 h (1 h: *p* < 0.01, 2 h: *p* < 0.001) after which their Tb began to drop. By 6 h p.i. with LPS, Tb for both NRS (*p* < 0.05) and PMN (*p* < 0.001) groups was significantly lower compared to their respective PBS treated controls. In comparison to these controls, NRS pre-treated mice remained hypothermic from 10 h p.i. - 22 h p.i. (*p* < 0.001) and by 23 h p.i. their Tb had completely returned to values of PBS-injected controls. However, PMN pre-treated mice maintained a hypothermic status for the duration of the experiment from 10 h p.i.-24 h p.i. (*p* < 0.001) compared to the PBS treated counterparts. Pre-treatment with PMN exacerbated the hypothermia and the initial drop in Tb at 6 h p.i. was more severe compared to their NRS+LPS counterparts (*p* < 0.01). Indeed, from 10 h p.i.-24 h p.i. Tb of PMN+LPS treated mice remained lower than NRS+LPS mice with the difference in Tb increasing over time (range: *p* < 0.01- *p* < 0.001). Baseline recordings did not show differences between groups except for a small increase in Tb following the inoculation with PMN lasting for approximately 2 h (*p* < 0.001) before returning to normal (**Supplementary Figures 4A, C**). The small increase in Tb was most likely caused by a brief inflammatory event associated with the initial break down of NGs.

Locomotor activity of both NRS and PMN pre-treated, PBS injected control mice showed a typical circadian day-night rhythm with low activity in the day and increased activity at night as well as a short-lasting peak due to the stress of handling and the injection while groups treated with LPS had almost completely depressed locomotor activity (**Figure 1B**). Immediately following treatment, both NRS and PMN groups had a main effect of LPS and showed a dramatic decrease in locomotor activity extending from 1 h p.i.-22 h p.i. (1 h, 3 h-22 h: *p* < 0.001; 2 h: *p* < 0.05). A fleeting impact of PMN did occur between 2 h p.i.-4 h p.i. (2 h: *p* < 0.01, 3 h: *p* < 0.05) regardless of LPS treatment where mice treated with PMN may have had increased locomotor activity but significant differences between NRS and PMN pre-treated groups were not detected. Baseline recordings did not show differences between groups except for a brief reduction in locomotor activity following the inoculation with PMN (*p* < 0.05) before returning to counterpart control levels (**Supplementary Figures 4B, D**).

Treatment with LPS had a strong effect on food and water intake at 24 h p.i. (**Figures 1C, D**). Both NRS and PMN pre-treated mice consumed negligible amounts of food (*p* < 0.001) and water (*p* < 0.001) regardless of pre-treatment with NRS or PMN. As a result, there was an LPS-induced decrease in body weight that was observed as early as 4 h p.i. for both LPS groups (*p* < 0.001). By 24 h p.i., the NRS (*p* < 0.001) and PMN (*p* < 0.001) pre-treated mice that received LPS weighed significantly less than their PBS treated counterparts (**Supplementary Figures 4E, F**). No differences were detected between the mice pre-treated with NRS or PMN in either the PBS or LPS groups.

### 3.3 Peripheral inflammatory mediators were exacerbated in neutrophil granulocyte depleted mice after high-dose-LPS-stimulation

**Figure 2:**
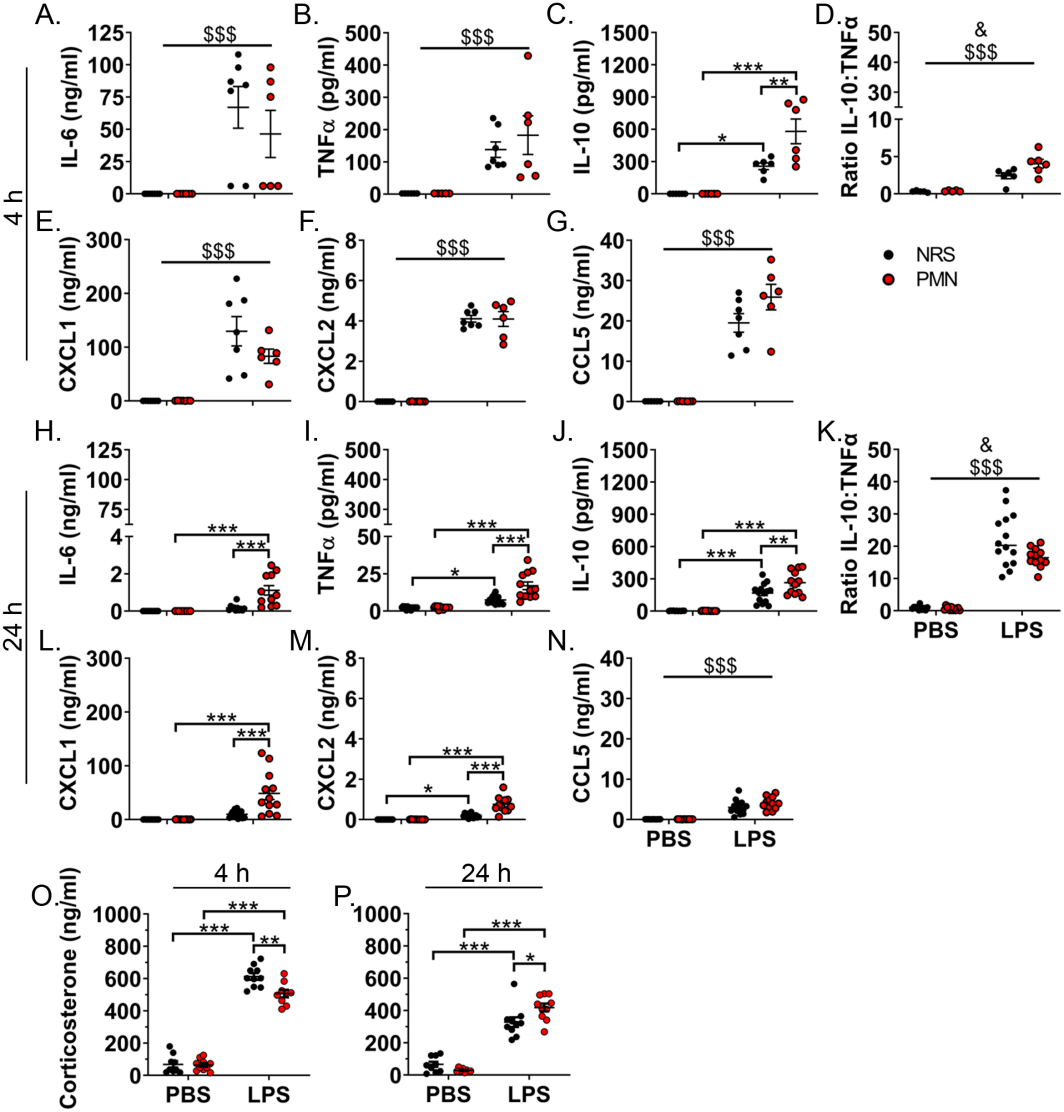
Peripheral inflammation was enhanced and corticosterone production altered by neutrophil granulocyte depletion during LPS-induced inflammation. Circulating levels of inflammatory mediators in the serum were compared between PMN or NRS pre-treated mice after PBS or LPS IP injection (2.5 mg/kg) 4 h and 24 h after stimulation. **(A, H)** Interleukin (IL)-6. **(B, I)** Tumor necrosis factor (TNF)α. **(C, J)** IL-10. **(D, K)** Ratio of IL-10:TNFα. **(E, L)** CXCL1. **(F, M)** CXCL2. **(G, N)** CCL5. **(O, P)** Corticosterone. Data are presented as dotplots with mean ± SEM (n=5–7 [4 h p.i.] / n=10–14 [24 h p.i.]). Statistical analysis was performed by Two-way ANOVA and Tukey *post-hoc* test with the main effects: ^&^PMN, ^$^LPS (*p< 0.05, **p< 0.01, ***p< 0.001, ^&^ *p*< 0.05, ^$$$^ *p*< 0.001).

The influence of NGs on inflammation in the periphery and the humoral pathway of immune-to-brain communication was assessed via circulating mediators indicative of the peripheral inflammatory response (**Figure 2**). NRS and PMN pre-treatment did not induce differences in circulating cytokine levels in PBS-inoculated control mice. The cytokines: IL-6, TNFα, IL-10; NG chemoattractants: CXCL1, CXCL2, CCL5; and corticosterone were, for the most part, increased at both time points after 2.5 mg/kg LPS treatment. As early as 4 h p.i., an exacerbated production of IL-10 (*p* < 0.01) was detectable in PMN pre-treated mice that received LPS compared to their NRS counterparts (**Figure 2C**). The IL-10:TNFα ratio, which served as another indicator of the inflammatory status (53), also showed a main effect of PMN that indicates an increase in the ratio for PMN pre-treated groups (*p* < 0.05) (**Figure 2D**). Moreover, also at 4 h p.i., circulating levels of corticosterone were lower in PMN pre-treated mice that received LPS (*p* < 0.01) compared to NRS pre-treated LPS stimulated counterparts (**Figure 2O**). Exacerbation of LPS-induced inflammatory mediators was more prevalent at 24 h p.i. at which time IL-6 (*p* < 0.001), TNFα (*p* < 0.001), IL-10 (*p* < 0.01), CXCL1 (*p* < 0.001), CXCL2 (*p* < 0.001), and corticosterone (*p* < 0.05) were all elevated in those mice pre-treated with PMN compared to their NRS counterparts (**Figured 2H-N,P**).

### 3.4 Neutrophil granulocyte recruitment to the brain was depressed in a structure dependent manner by anti-polymorphonuclear serum

**Figure 3:**
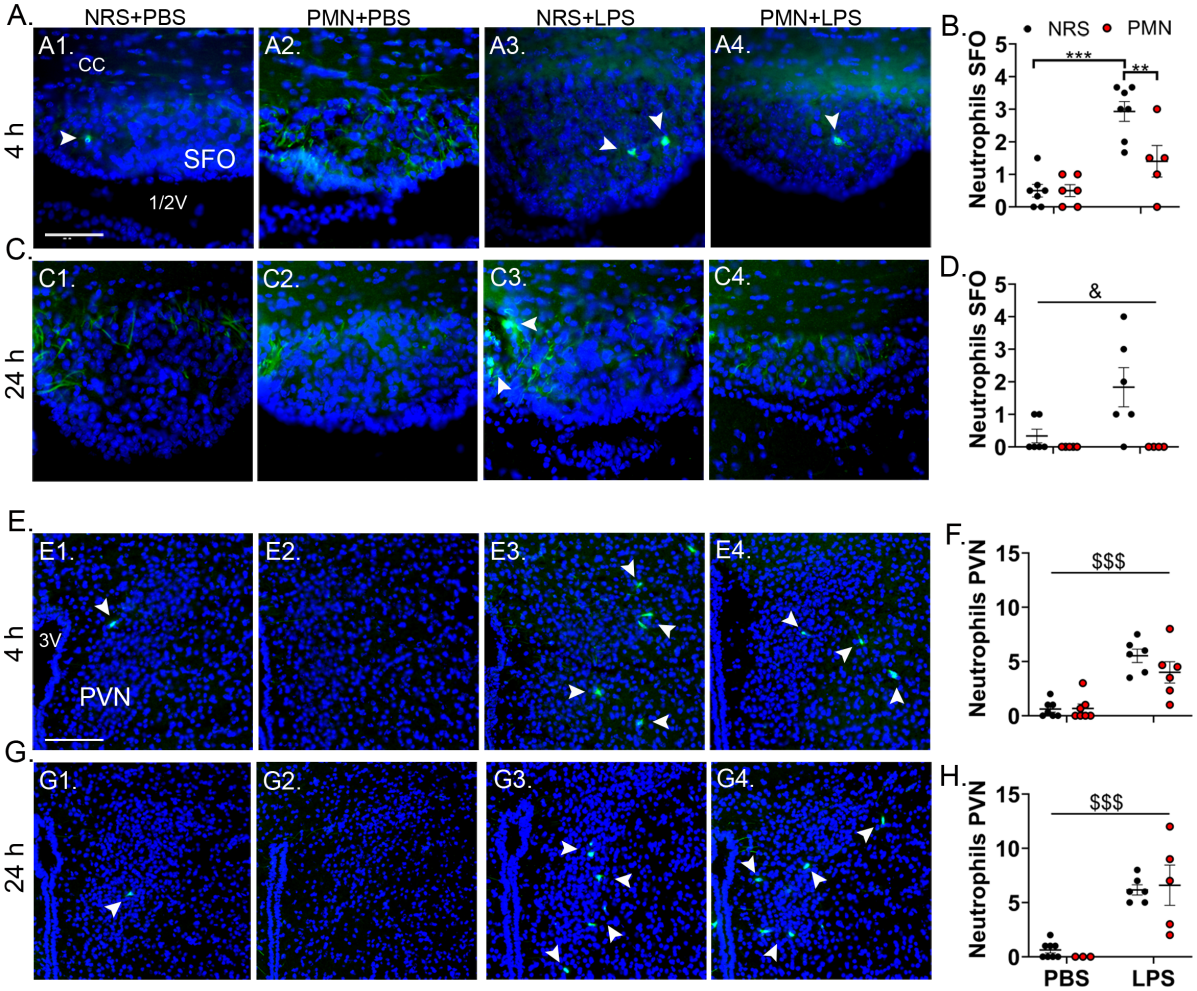
LPS-induced neutrophil granulocyte recruitment to the brain was attenuated in a structure dependent manner by pre-treatment with PMN but not NRS. **(A, C)** Immunofluorescence staining of neutrophil granulocytes (green) at the level of the subfornical organ (SFO) 4 h or 24 h after stimulation (scale bar = 50 μm). **(B, D)** The number of neutrophil granulocytes in the SFO were counted and compared between PMN or NRS pre-treated mice after PBS or LPS IP injection 4 h or 24 h after stimulation. Data are presented as dotplots with mean ± SEM (n=4-7). **(E, G)** Immunofluorescence staining of neutrophil granulocytes (green) at the level of the paraventricular nucleus (PVN) for the indicated time point (scale bar = 100 μm). **(F, H)** The number of neutrophil granulocytes in the PVN were counted and compared between PMN or NRS pre-treated mice after PBS or LPS IP injection 4 h or 24 h after stimulation. Data are presented as dotplots with mean ± SEM (n=3-8). The corpus callosum (cc) and 1/2/3 ventricle (v) are shown as structural reference points. Dapi (blue) visualizes the surrounding tissue. Statistical analysis were performed by Two-way ANOVA and Tukey *post-hoc* test with the main effects: ^&^PMN, ^$^LPS (**p< 0.01, ***p< 0.001, ^&^ *p*< 0.05, ^$$$^ *p*< 0.001).

Immunofluorescence staining of MPO at the level of the SFO and PVN was used to investigate NGs role in cellular communication within brain structures where recruitment has previously been shown (**Figure 3**) (33, 48). NG recruitment was overall absent or only one NG was present in both NRS and PMN pre-treated groups after PBS inoculation. However, following 2.5 mg/kg LPS treatment, NG recruitment to the SFO was increased in NRS mice at 4 h p.i. (*p* < 0.001) but not 24 h p.i. (**Figures 3A, B**). Pre-treatment with PMN effectively depleted NG recruitment to the SFO 4 h p.i. (*p* < 0.01) and a main effect of PMN at 24 h p.i. may indicate overall reduced NG recruitment (*p* < 0.05) (**Figured 3C, D**). At the level of the PVN, there was a strong effect of LPS that increased NG recruitment, regardless of pre-treatment with NRS or PMN, at both time points (4 h & 24 h: *p* < 0.001) (**Figures 3E-H**). The PMN-effect observed at the SFO was absent at the PVN. In summary, PMN was able to attenuate NG recruitment to the brain after LPS-stimulation in a structure-dependent manner.

### 3.5 Hypothalamic inflammation was exacerbated after high-dose-LPS-stimulation

**Figure 4:**
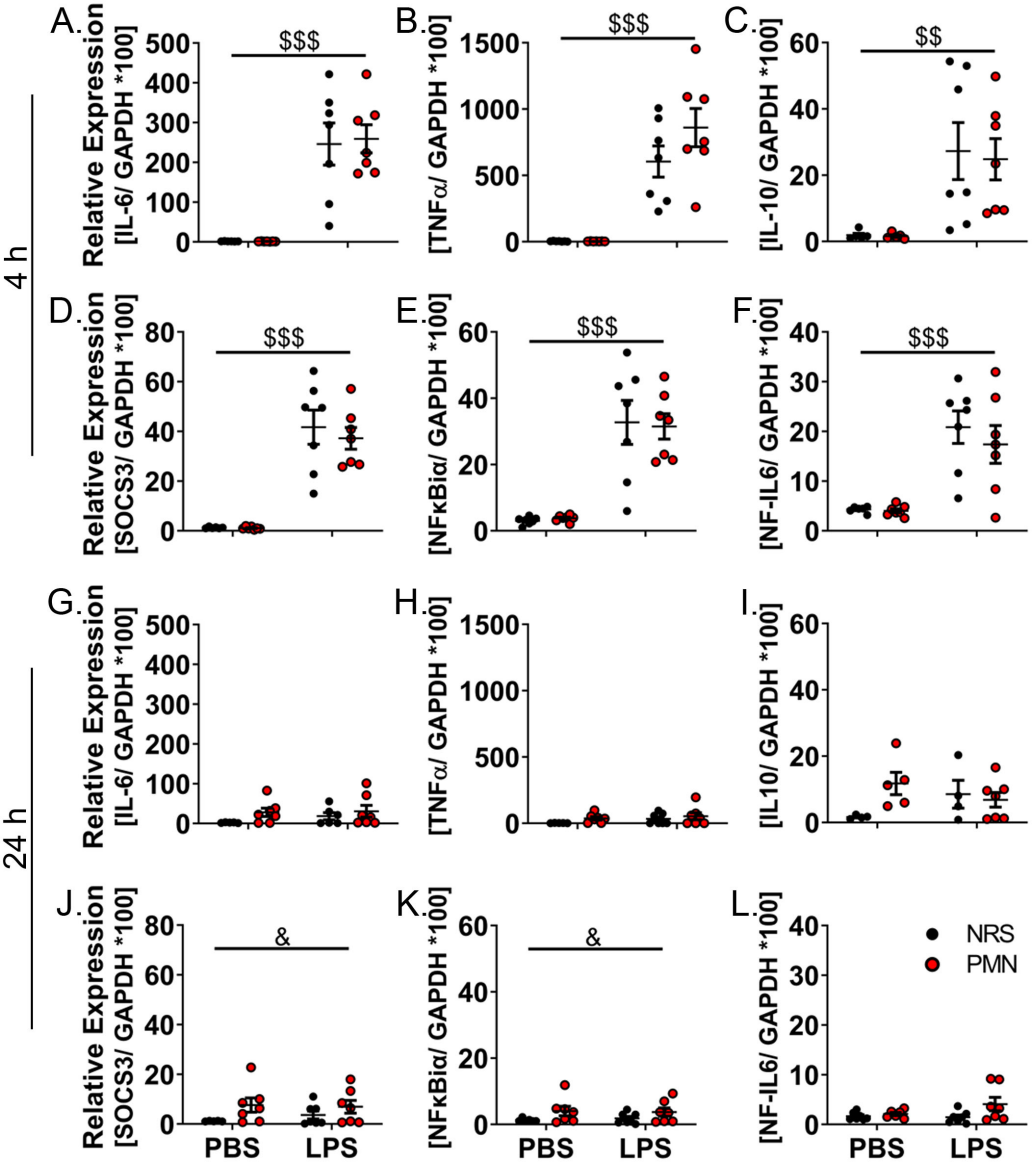
LPS-induced expression of inflammatory signaling pathways in the hypothalamus was altered by neutrophil granulocyte depletion 24 h after LPS-stimulation. RT-qPCR analysis of hypothalamic inflammation was compared between PMN or NRS pre-treated mice after PBS or LPS IP injection 4 h or 24 h after stimulation. **(A, G)** IL-6. **(B, H)** TNFα. **(C, I)** IL-10. **(D, J)** Suppressor of cytokine signaling 3 (SOCS3). **(E, K)** Nuclear factor κB inhibitor α (NFκBiα). **(F, L)** Nuclear factor (NF)-IL6. Data are presented as dotplots with mean ± SEM (n=4-7). Statistical analysis were performed by Two-way ANOVA with the main effects: ^&^PMN, ^$^LPS (^&^ *p*< 0.05, ^$$^ *p*< 0.01, ^$$$^ *p*< 0.001).

To investigate how peripheral inflammation could induce inflammation in the brain and the generation of sickness responses, we evaluated the expression of inflammatory target genes in the hypothalamus (**Figure 4**). Hypothalamic mRNA expression of the cytokines IL-6, TNFα, and IL-10 were assessed. There was, once again, a strong effect of LPS treatment at 4 h p.i. that increased the expression of all cytokines regardless of pre-treatment with NRS or PMN (IL-6: *p* < 0.001, TNFα: *p* < 0.001, IL-10: *p* < 0.01); no effects of PMN were observed (**Figures 4A-C**). By 24 h p.i. the LPS-induced increase in cytokine expression was no longer present and no effects of PMN were observed (**Figures 4G-I**).

Markers for different signaling pathways including the STAT3 activation marker suppressor of cytokine signaling 3 (SOCS3) (32, 48), the nuclear factor (NF) κB activation marker NFκB inhibitor α (-iα), and nuclear factor IL-6 (NF-IL6) (48, 54) were also assessed. The mRNA-expression for SOCS3 (*p* < 0.001), NFκBiα (*p* < 0.001), and NF-IL6 (*p* < 0.001) were increased by treatment with 2.5 mg/kg LPS at 4 h p.i. regardless of pre-treatment with NRS or PMN; no effects of PMN were observed (**Figures 4D-F**). By 24 h p.i. the LPS-induced increase in expression was no longer present for any of the signaling markers but SOCS3 (*p* < 0.05) and NFκBiα (*p* < 0.05) did show a main effect of PMN that indicated an increased expression regardless of LPS treatment (**Figures 4J-L**). However, further analysis of STAT3 signaling by immunohistochemical staining at the level of the OVLT failed to detect any differences between groups pre-treated with NRS or PMN (**Supplementary Figure 5**).

Cellular immune-to-brain communication was further evaluated for NGs using the chemoattractant, CXCL1 and neutrophil elastase (ELANE), as well as the activated microglial and perivascular macrophage markers CD68 and CD163, respectively (**Supplementary Figure 6**). Fitting with the immunofluorescence staining, which showed increased NG recruitment to the brain (**Figure 3**), CXCL1 (*p* < 0.001) and ELANE (*p* < 0.001) were both elevated in an LPS-dependent manner with no observed effects of PMN (**Supplementary Figures 6A, B**). By 24 h p.i. the LPS-induced increase in both NG markers expression was no longer present for either group (**Supplementary Figures 6E, F**). In contrast, CD68 and CD163 were not increased by LPS treatment at either time point and were also unaffected by pre-treatment with PMN (**Supplementary Figures 6C, D, G, H**).

### 3.6 Prostaglandin E2 synthesis in the hypothalamus was exacerbated after high-dose-LPS-stimulation

**Figure 5:**
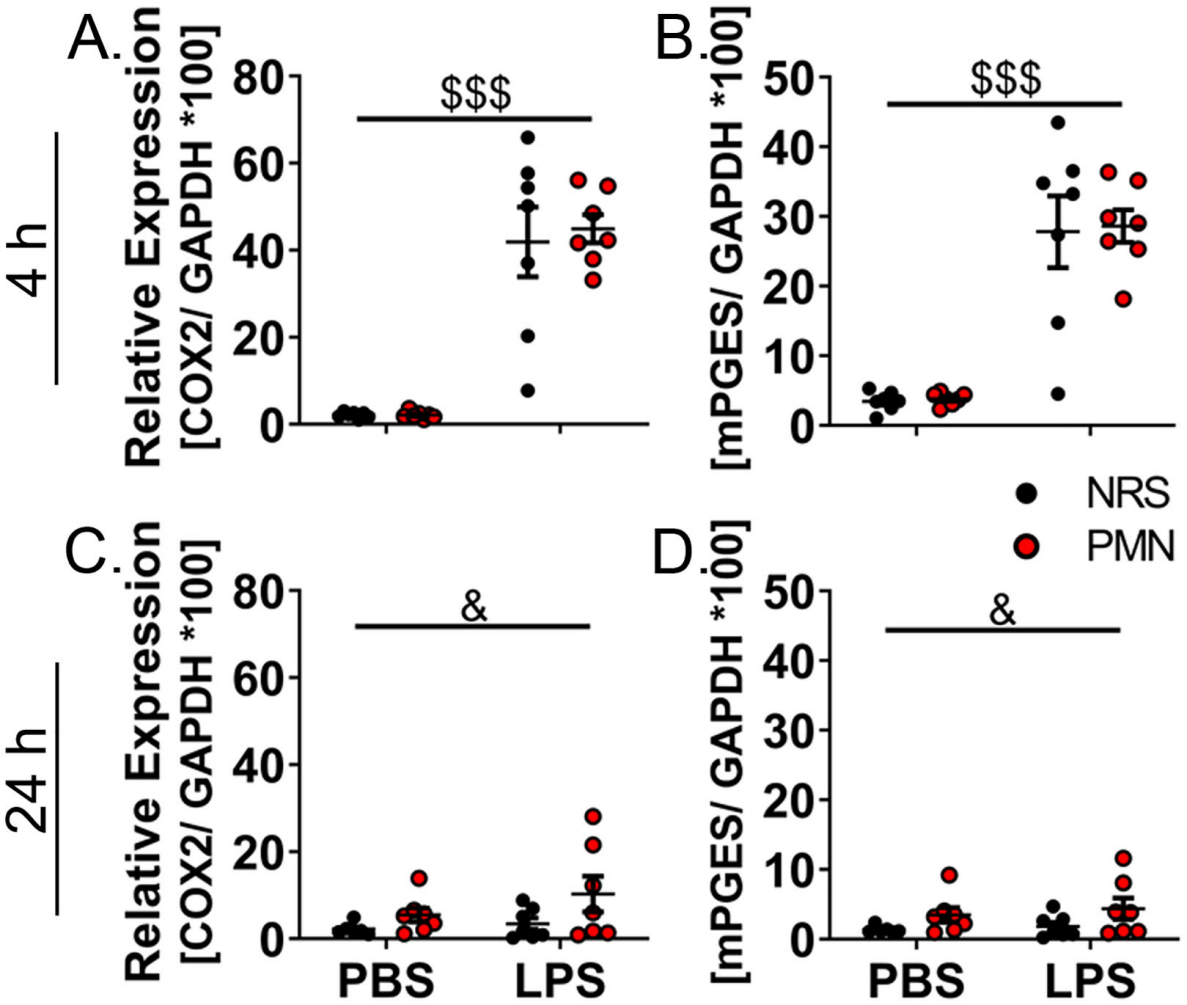
Expression of prostaglandin E2 catalyzing enzymes was altered by neutrophil granulocyte depletion 24 h after PBS- or LPS-stimulation. RT-qPCR analysis of hypothalamic prostaglandin E2 enzymes were compared between PMN or NRS pre-treated mice after PBS or LPS IP injection 4 h or 24 h after stimulation. **(A, C)** Cyclooxygenase 2 (COX2). **(B, D)** Microsomal prostaglandin E synthase (mPGES). Data are presented as dotplots with mean ± SEM (n=6-7). Statistical analysis were performed by Two-way ANOVA with the main effects: ^&^PMN, ^$^LPS (^&^ *p*< 0.05, ^$$$^ *p*< 0.001).

Prostaglandin E2 (PGE2) is an important mediator of inflammation that can also act as an endogenous pyrogen and contribute to fever generation or other thermoregulatory processes (55, 56). Therefore, mechanisms of PGE2-synthesis could be used as indicators in the development of sickness responses (33, 48, 56). Using the rate-limiting enzymes cyclooxygenase 2 (COX2) and microsomal prostaglandin E synthase (mPGES) we assessed mRNA-expression levels in the hypothalamus (**Figure 5**). The expression for COX2 (*p* < 0.001) and mPGES (*p* < 0.001) were increased by treatment with 2.5 mg/kg LPS at 4 h p.i. regardless of pre-treatment with NRS or PMN; no effects of PMN were observed (**Figures 5A, B**). By 24 h p.i. the LPS-induced increase in expression was no longer present for either COX2 or mPGES but, as was observed for inflammatory signaling (**Figure 4**), a main effect of PMN did indicate an increase in expression regardless of LPS treatment for each enzyme (*p* < 0.05) (**Figures 4C, D**). Exacerbated PGE2-synthesis in PMN pre-treated mice could contribute to the altered thermoregulatory response during high-dose-LPS-stimulation but, overall, mechanisms of PGE2 synthesis were not significantly affected by PMN pre-treatment.

### 3.7 IL-6, CXLC2, and CCL5 were exacerbated in the hypothalamus of neutrophil granulocyte depleted mice after high-dose-LPS-stimulation

**Figure 6:**
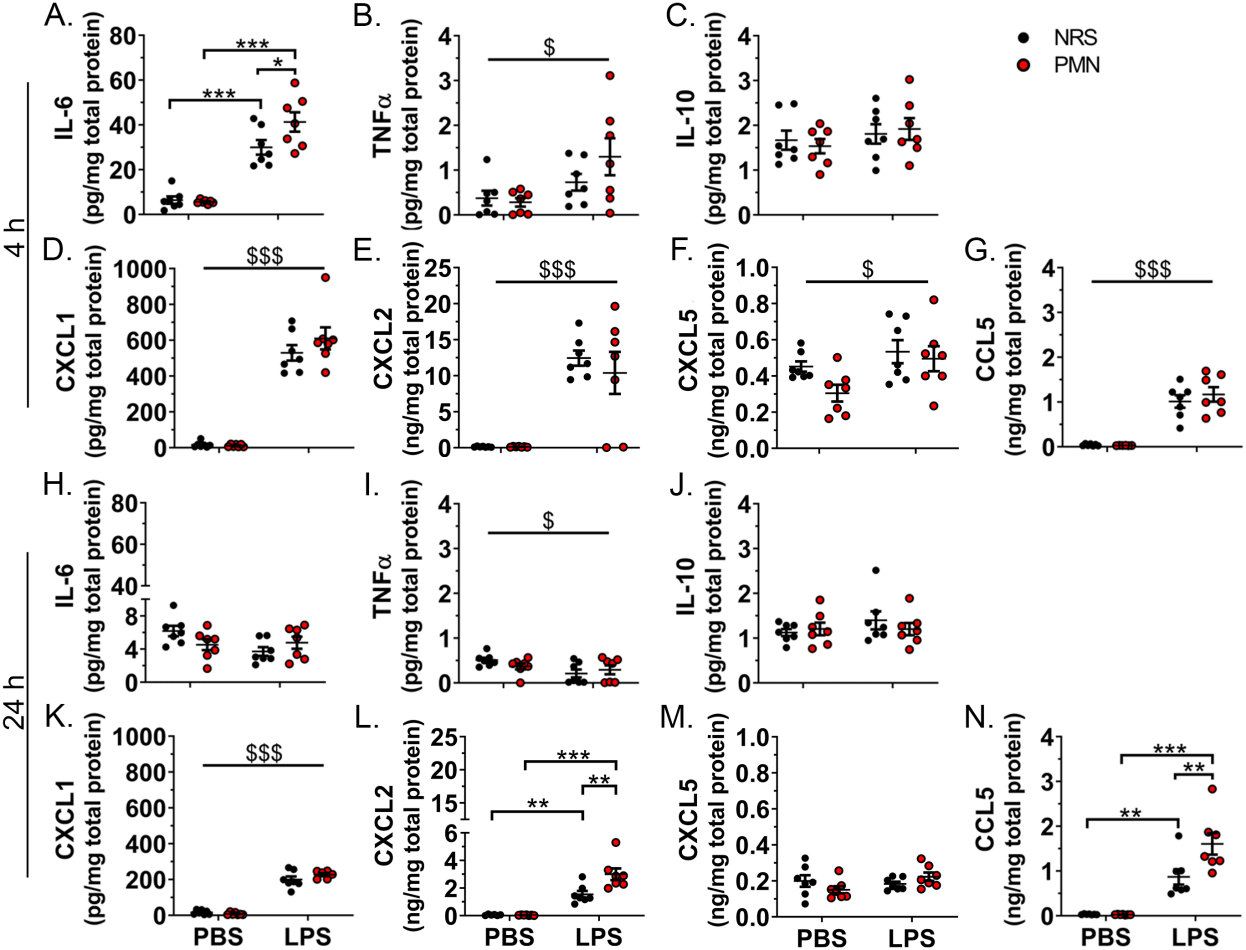
LPS-induced IL-6, CXCL2, and CCL5 levels in the hypothalamus were exacerbated by neutrophil granulocyte depletion. Hypothalamic levels of inflammatory mediators were compared between PMN or NRS pre-treated mice after PBS or LPS IP injection 4 h or 24 h after stimulation. **(A, H)** IL-6. **(B, I)** TNFα. **(C, J)** IL-10. **(D, K)** CXCL1. **(E, L)** CXCL2. (**F, M**) CXCL5. (**G, N**) CCL5. Data are presented as dotplots with mean ± SEM (n=6-7). Statistical analysis were performed by Two-way ANOVA and Tukey *post-hoc* test with the main effect: ^$^LPS (*p< 0.05, **p< 0.01, ***p< 0.001, ^$^ *p*< 0.05, ^$$$^ *p*< 0.001).

To further investigate indicators that mRNA expression of inflammatory mediators in the hypothalamus may have been enhanced in neutrophil depleted mice, we used a magnetic Luminex assay to confirm these results on the protein level (**Figure 6**). Indeed, we saw that several cytokines were not only increased by treatment with 2.5 mg/kg LPS at 4 h p.i. but were exacerbated in the PMN pre-treated mice. Of the cytokines measured, IL-6 levels were significantly increased by LPS-induced inflammation in NRS (*p* < 0.001) and PMN (*p* < 0.001) pre-treated mice (**Figure 6A**). TNFα (*p* < 0.05), CXCL1 (*p* < 0.001), CXCL2 (*p* < 0.001), CXCL5 (*p* < 0.05), and CCL5 (*p* < 0.001) all had main effects of LPS that indicated increased overall cytokine levels regardless of pre-treatment with NRS or PMN (**Figures 6B, D–G**); no effects of PMN were observed. For IL-6, the LPS-induced increase was intensified in those mice pre-treated with PMN (*p* < 0.05) (**Figure 6A**). Though moderate, the enhanced cytokine production does suggest that PMN pre-treated mice were experiencing a higher degree of inflammation in the brain at this time point.

As most cytokine levels dropped or returned to basal levels, by 24 h p.i. fewer PMN-dependent differences could be detected (**Figure 6H-N**). TNFα (*p* < 0.05) still had a minor LPS effect indicating a decrease in cytokine levels overall but had returned to approximately basal levels (**Figure 6I**). The values for CXCL1 (*p* < 0.001) meanwhile, remained elevated in an LPS-dependent manner regardless of pre-treatment with NRS or PMN (**Figure 6K**). Finally, the LPS-induced increase in CXCL2 and CCL5 (NRS: *p* < 0.01, PMN: *p* < 0.001) was now significantly intensified in those mice pre-treated with PMN (*p* < 0.01) (**Figures 6L, N**).

### 3.8 NET formation in the hypothalamus may contribute to the sickness response after high-dose-LPS-stimulation

**Figure 7:**
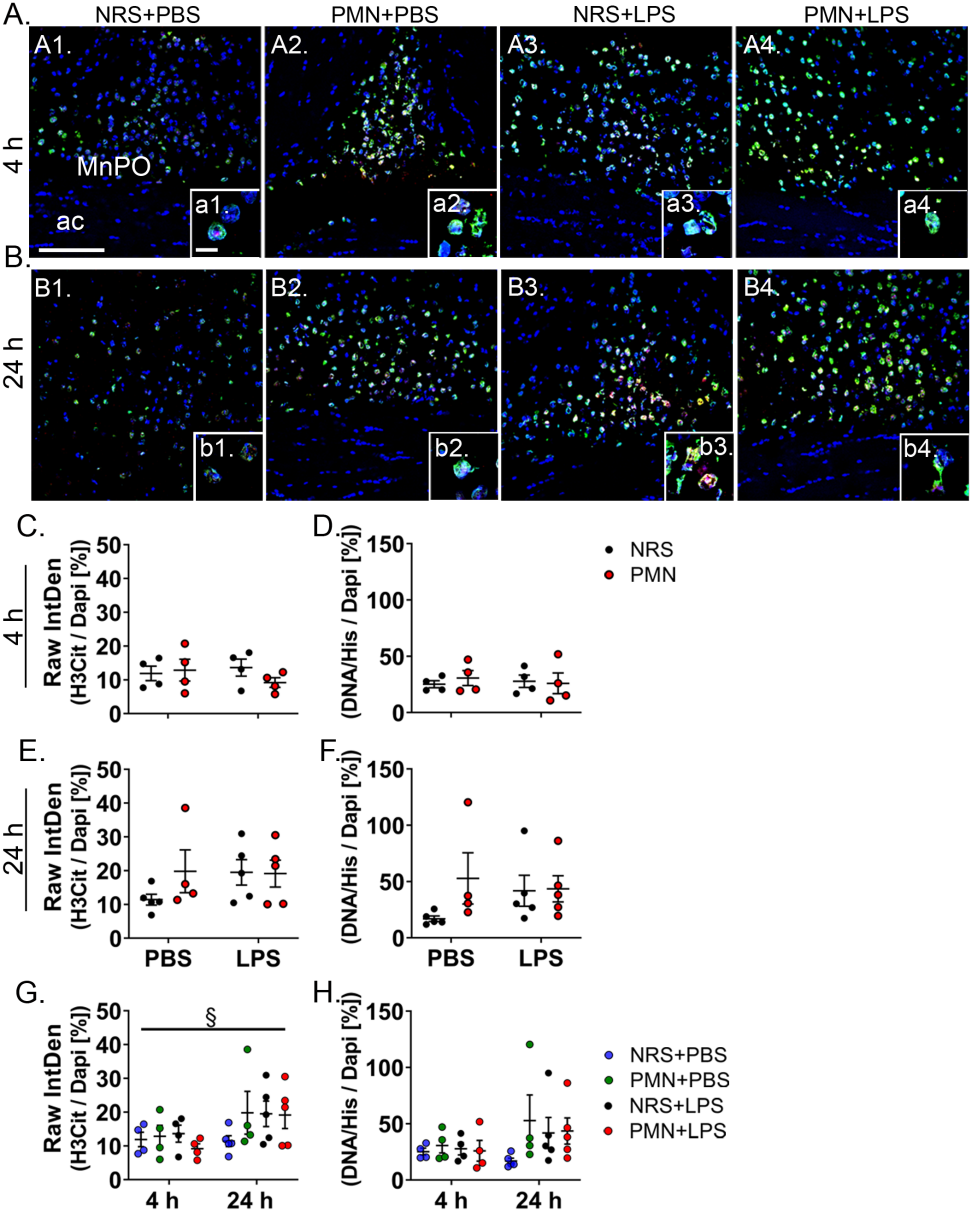
Neutrophil extracellular trap (NET) formation in the brain was not affected by neutrophil granulocyte depletion. **(A, B)** Immunofluorescence staining of the NET markers citrullinated histone H3 (H3cit; red) and DNA/histone complex (DNA/His; green) at the level of the median preoptic nucleus (MnPO) 4 h or 24 h after stimulation (overview scale bar = 25 μm, insert scale bar = 10 μm). The arcuate nucleus (ac) is shown as a structural reference point. Dapi (blue) visualizes the surrounding tissue. **(C, E)** Raw integrated density (Raw IntDen) for H3Cit in relation to Dapi were compared between PMN or NRS pre-treated mice after PBS or LPS IP injection 4 h or 24 h after stimulation. **(D, F)** Raw IntDen for DNA/His in relation to Dapi were compared between PMN or NRS pre-treated mice after PBS or LPS IP injection 4 h or 24 h after stimulation. **(G)** Raw IntDen for H3Cit in relation to Dapi were compared over time. **(H)** Raw IntDen for DNA/His in relation to Dapi were compared over time. Data are presented as dotplots with mean ± SEM (n=4-5). Statistical analysis were performed by Two-way ANOVA with the main effects: ^§^Time (^§^ *p*< 0.05).

Since NET formation in the brain has been associated with exacerbated inflammation in a model of LPS-induced inflammation (57), we investigated if NETosis in the hypothalamus could be a contributing factor for the exacerbated sickness responses observed in neutrophil-depleted mice after treatment with 2.5 mg/kg LPS. Looking at the level of the MnPO, a hypothalamic structure known to contribute to fever induction pathways (58, 59), we analyzed the ratios of our two NET markers H3Cit or DNA/His to nuclear Dapi stain (**Figure 7**). At 4 h p.i. (**Figures 7A, C, D**) and 24 h p.i. (**Figures 7B, E, F**) there were no effects of LPS or PMN. Though, when compared overtime (**Figures 7G, H**), the H3Cit: Dapi ratio was significantly increased at 24 h p.i. regardless of treatment group (**Figure 7G**). Together, these data suggest that during LPS-induced inflammation NET formation may increase overtime but is unaffected by pre-treatment with NRS or PMN.

## 4 Discussion

Our investigation into the role of NGs during high-dose-LPS-stimulation is a novel examination of the potential anti-inflammatory properties of NGs in modulating peripheral inflammation, brain inflammation, and sickness behavior. We showed that partial NG depletion not only enhanced inflammation but also exaggerated the brain-controlled hypothermic response. Indeed, peripheral cytokine profiles were enhanced by PMN during inflammation and attenuated LPS-induced NG recruitment to the SFO was accompanied by exacerbated hypothalamic IL-6, CXCL2, and CCL5 in neutropenic mice. These mice also experienced impaired activation of the hypothalamic-pituitary-adrenal (HPA) axis that resulted in a dampened but prolonged response. Together, these results indicate that during severe inflammation NGs, directly or indirectly, contribute to an anti-inflammatory profile and are significant mediators of sickness responses.

Previous studies have reported associations between diminished NG activity, as experienced by neutropenic mice, and the severity of inflammation (9, 60–62). In our own experimental model, we saw a clear exacerbation of sickness responses in neutropenic mice in the absence of an infection with live bacteria. While both groups became hypothermic after treatment with LPS, the drop in the Tb of immunocompetent mice (pre-treated with NRS) was consistent with the existing literature (50, 63, 64), whereas the drop in Tb of neutropenic mice was more pronounced. Hypothermia associated with inflammation may represent a host defense mechanism (65) and indeed, inhibition of hypothermia during severe systemic inflammation in mice and rats has even been shown to increase mortality (66–70). Romanovsky and colleagues have shown that hypothermia like fever is a defense mechanism, which is induced in the brain (66). Indeed, findings suggest that neurons in the DMH/ventromedial hypothalamic nucleus are important for induction of LPS-induced hypothermia by cold-seeking behavior (71, 72). However, ineffective thermoregulation is a strong indicator of disease severity where hypothermic patients notoriously suffer from higher mortality rates in comparison to patients that develop fever (73–76). One possible driving force in the development of hypothermia is an exaggerated peripheral inflammatory response. It has been shown that, through interactions at CVOs or at the BBB via endothelial cells, LPS, IL-6, and TNFα (77–80) are able to activate glial cells, signaling pathways, and promote the local production of inflammatory mediators at thermosensitive regions of the hypothalamus (81–85). Indeed, overproduction of circulating levels of IL-6, TNFα, and IL-10 were accompanied by the development of hypothermia during different animal models of sepsis (63, 86–88). Significant alterations to peripheral cytokines could have broad implications for the humoral pathway of immune-to-brain communication, in particular, during septic-like inflammation due to increased permeability of the BBB (89, 90). In accordance with previous studies, IL-6, TNFα, and IL-10 were elevated in both groups by LPS treatment (26, 91) but circulating levels were exacerbated in neutropenic mice in the absence of enhanced bacterial growth.

Important markers of sepsis severity include IL-6 and TNFα. Both cytokines can promote excessive inflammation (92, 93) and, in the case of TNFα, have been associated with increased mortality (94, 95). Circulating levels of IL-6 are commonly elevated in neutropenic patients with sepsis, as we observed in our experiment, and are associated with more severe outcomes (93, 96). In comparison, despite associations linking high levels of TNFα with increased mortality, its exact role remains elusive as no direct connection between TNFα and sepsis severity has been made (95, 97). The more complex peripheral role of TNFα, as with IL-6, can nevertheless also serve as a humoral mediator in immune-to-brain communication and regulate sickness behavior through pro-inflammatory actions (85). During severe LPS-induced inflammation, IL-6 and TNFα disrupt tight junctions and promote a loss of endothelial cell integrity, which contribute to the BBB breakdown (98, 99). A consequence of the weakened BBB is an increased interaction of circulating cytokines with the brain (89, 90). In fact, by acting through TNF receptor 1 (TNFR1), TNFα in the brain can significantly influence the development of sepsis associated encephalopathy (SAE) and mediate certain NG functions (100). An enhanced expression of TNFα and TNFR1 has already been documented in the preoptic area of mice during septic inflammation by Mul Fedele and colleagues (2020). They found that severe hypothermia was associated with higher circulating levels of TNFα and knocking-out TNFR1 improved survival rates in mice (88). Despite improved survival rates, the persistence of hypothermia in TNFR1-deficient mice indicates that additional signaling is also necessary for development of the thermoregulatory response. Indeed, there is compelling evidence that interactions between TNFα and IL-6 can shift TNFα from a pyrogen to a cryogen (101, 102). Together, the existing literature, as well as the present experiment, suggests that the inflammatory mediators TNFα and IL-6 can exacerbate hypothermia in neutropenic mice.

In addition to the elevated levels of TNFα and IL-6, we also observed that IL-10 was enhanced by neutropenia during LPS-induced inflammation at both time points investigated. As an anti-inflammatory cytokine, IL-10 modulates inflammation but, during sepsis, this effect can be detrimental since a dampened immune response can exacerbate disease progression (103). Increased IL-10 levels have been previously detected in neutropenic patients, and could be the consequence of insufficient NG actions as the first line of defense that contribute to an overall weakened inflammatory response (104). Alterations to other immune cells can also induce a cytokine shift and monocytes, a major source of IL-10, can undergo leukocyte reprogramming or develop monocyte anergy during sepsis leading to elevated levels of this important cytokine (105). Together, these alterations contribute to an immunosuppressive status during sepsis. While the known anti-inflammatory capacity of IL-10 in neutropenic mice reflects pro-inflammatory potential of NGs, enhanced circulating IL-10 levels during the course of inflammation may as well reflect the course of a preceding exacerbated inflammatory response. Indeed, whether due to an exacerbation of septic symptoms and shift in cell function or merely a neutralization of the stronger pro-inflammatory response, the fact that neutropenic mice maintained elevated levels of IL-10 at both time points served as an indicator of immunosuppression and disease severity after the systemic LPS-challenge (103, 106–108). Interestingly, we were previously able to show that acute neutralization of circulation IL-10 by an antiserum inhibited LPS-induced hypothermia in severely inflamed rats (109) suggesting that IL-10 may actually convey exacerbation of hypothermia in neutropenic mice observed in our present study. Moreover, Steiner and colleagues (2013) revealed evidence for a potential peripheral action of LPS to induce hypothermia. When LPS was injected into the ventricles of mouse brains (intracerebroventricularly), it evoked fever, whereas LPS doses of a similar magnitude injected i.p. induced a hypothermic response (110). Such data overall supports a peripheral action of neutropenia to elicit enhanced systemic inflammation and IL-10 mediated (109) exacerbated hypothermia.

Dysregulation of immune responses during septic-like inflammation can also include the generation of glucocorticoids via the HPA-axis. Under typical circumstances, glucocorticoids (corticosterone rodents; cortisol humans) perform modulatory functions that suppress the immune response and contribute to restored homeostasis (111–113). The disruption of these functions and reduced glucocorticoid metabolism can increase circulating levels, as has been shown in the plasma of critically ill ICU patients (114). Our findings were consistent with those of the existing literature as we found that circulating corticosterone levels were increased by LPS-induced inflammation in neutropenic mice. However, additional comparisons revealed that neutropenic mice experienced dampened but prolonged activation of their HPA-axis. Reduced corticosterone levels could represent an insufficient response during early inflammation (4 h), possibly due to an inadequate number of NGs that may contribute to the increase in circulating inflammatory mediators. These cytokines could subsequently signal cells of the CVOs or reach other brain structures, due to disruption of the BBB, to boost HPA activation as has been previously described (115, 116). Although initially beneficial, excessive corticosterone production has been associated with death in rats after induction of sepsis using a model of cecal ligation and puncture (CLP). Indeed, high corticosterone/cortisol levels can be a predictor of mortality in rats and humans diagnosed with septic shock (117, 118). Ultimately, disruption of the HPA-axis experienced by neutropenic mice could be another indicator, cause or consequence of the severe inflammation induced by a high dose of LPS.

Since we were particularly interested in the role that NGs could play in the development of brain inflammation and SAE, we also assessed NG recruitment to the brain using the SFO, a sensory CVO, and the PVN, a pivotal brain structure for HPA-axis activation. Having already seen enhanced circulating levels of CXCL1 and CXCL2 in neutropenic mice in comparison to the immunocompetent counterparts during LPS-induced inflammation, we were interested if chemokine production in the brain was also altered in neutropenic mice. If, as we hypothesize, NG-to-brain communication modulates inflammatory mediators, then local production of chemoattractants in the hypothalamus could be indicators of recruitment. For instance, by producing CXCL1, brain endothelial cells enhance intercellular adhesion molecule (ICAM)1 mediated NG binding and ELANE eases passage through the endothelium (119–121). In the present experiment, the expression of CXCL1 and ELANE were both increased in the hypothalamus during LPS-induced inflammation regardless of neutropenic status but only at the early time point (4 h). These observations match our immunofluorescent data, which also revealed a significant recruitment of NGs at 4 h p.i. to the SFO and the PVN. Although pre-treatment with PMN only reduced recruitment to the SFO, neuronal projections to the PVN from the SFO (122) could be activated by NGs and may support a potential pathway for NG modulation of the HPA-axis and a possible reason why neutropenic mice experienced dysregulation of this axis.

In addition, damage to the BBB by an exaggerated immune response can augment interactions between the brain, circulating immune cells, and inflammatory mediators. Together, these interactions can increase cytokine generation in the brain, which seems to be particularly important for the development of SAE (30, 89, 90, 123). Further analysis of the hypothalamus was used to determine if neutropenia could alter cellular activation. The early increases in expression of IL-6, TNFα, and IL-10 could be explained by a combined action of peripheral and locally produced cytokines in immunocompetent and neutropenic mice. By analyzing the STAT3, NFκB, and NF-IL6 signaling pathways, we were able to assess the severity of inflammation in the brain, as has previously been shown (48, 124, 125). All three signaling pathways have some capacity for immune cell regulation and recruitment, specifically for NGs, e.g. through the expression and modulation of ICAM1 (126, 127). Though the strong LPS effect indicated increased expression for all signaling pathways at 4 h p.i., consistent with what has been previously shown (32, 48, 124, 125), the minor effects of pre-treatment with PMN at 24 h p.i. for SOCS3 and NFκBiα may indicate exacerbated pro-inflammatory signaling in the neutropenic mice. As a known activator of STAT3-signaling in the brain (128, 129), exacerbated LPS-induced hypothalamic levels of IL-6 in neutropenic mice further supports enhanced activation of the STAT3 pathway. In previous studies, IL-6 protein levels in the brain have also been shown to increase 4 h after i.p. injection of 10 μg/g LPS (130, 131), a dose that is in a similar range ~1 mg/kg as to the one used by us 2.5 mg/kg. Indeed, brain IL-6 is necessary to induce fever as previously revealed using cytokine deficient animals (132). During LPS-induced inflammation, IL-6 dependent signaling has been linked to the febrile response by way of STAT3-induced COX2 synthesis in, for example, brain endothelial cells (129, 133, 134). While we didn’t detect a difference in the immunoreactivity of STAT3 at the level of the OVLT between immunocompetent and neutropenic mice, we did find indications that hypothalamic synthesis of PGE2 may be enhanced by neutropenia at 24 h p.i. using the precursors COX2 and mPGES. Increased production of brain PGE2 is associated with severe inflammation, increased permeability of the BBB, and sickness responses (135, 136). Perivascular macrophages have also been identified as sources of PGE2 and stimulating PGE2 release from brain endothelial cells (137, 138). However, we were unable to detect increased expression of a marker protein for perivascular macrophages (CD163) suggesting that they did not significantly contribute to the altered expression in the enzymes for PGE2 synthesis. If IL-6-induced COX2 expression leads to an upregulation of hypothalamic PGE2 it may indicate a latent modulatory impact of NGs on PGE2 production and signaling during high-dose-LPS-induced inflammation.

Interestingly, besides IL-6, we also revealed significant exacerbation of CXCL2 levels in the hypothalamus of neutropenic mice 24 h but not 4 h after LPS-stimulation. Indeed, CXCL2 also known as macrophage inflammatory protein-2, has previously been shown to act as an endogenous circulating pyrogen during early LPS-induced fever (139). Miñano and colleagues (2004) revealed that acute depletion of CXCL2 by a polyclonal antibody inhibited the early phase of LPS-induced fever but not the prolonged fever response in cyclophosphamide-induced leukopenic rats. Our results for CXCL2 in plasma and the hypothalamus support some potential contribution to an enhanced prolonged inflammatory response in the brain but not in plasma of neutropenic mice.

Moreover, there is evidence that LPS-induced depression-like behaviors can be altered by NGs via NET formation (140) or direct NG-to-brain signaling (28). Since septic-like inflammation can increase NG recruitment to the brain (28, 41) and inhibition of NETs can attenuate SAE symptoms related to memory impairment, BBB integrity, and glial activation (141), the role of NETs in the development of SAE could be significant and warranted further investigation. Using the NG migration enzyme ELANE, which is also released during NETosis (120, 121), we already observed increased hypothalamic levels during LPS-induced inflammation. However, ELANE alone is not sufficient to evaluate NETosis so we also included immunofluorescent detection of the markers H3Cit and DNA/Histone at the MnPO. Despite a recent study that found increased NETs in the hippocampus of mice during a model of CLP (141), we did not detect any differences between groups. Since H3Cit density seemed to increase over time during severe inflammation in our study, NETs could contribute to time-dependent generation of sickness responses overall. In addition, while we have chosen the MnPO as a pivotal hypothalamic brain structure for thermoregulation during inflammation, other brain structures should be considered for assessments of NETs in future studies. We have previously detected NET markers for DNA-histone complexes at the level of the brain stem in rats during severe systemic inflammation (1 mg/kg, 8 h) (49), but a more comprehensive analysis of hypothalamic structures is still necessary to understand the extent by which NETs in the brain can modulate inflammation during septic-like inflammation.

Limitations for the present study include that the established model of neutropenia (24 h) using an anti-serum only partially depleted NGs by ~25%. While we did not detect any significant alterations for other leukocyte populations (eosinophil granulocytes, basophil granulocytes, monocytes, and lymphocytes) in hematological analyses of preliminary experiments at the dose of PMN applied in the present study, higher PMN doses did also significantly deplete basophil granulocytes. Moreover, there was a trend for additional non-selective effects at high doses of PMN. More comprehensive characterization was not feasible for the entire experimental cohort due to sample volume constraints and experimental design. In addition, although monocyte percentages were not significantly altered, high variability amongst the LPS-treated groups could suggest functional or compositional shifts in monocyte populations and, therefore, cannot be excluded as potential contributing factors to the observed effects in our present study and should be further investigated in the future. Attempts to more drastically deplete NGs were accompanied by increased mortality during LPS-induced severe systemic inflammation highlighting the functional significance of NG during systemic inflammation. To more robustly deplete NGs, an alternative approach would be genetic modified neutropenic mice (Csf3r-/-, https://www.jax.org/strain/017838). However, such chronic genetic modified models can be accompanied by long-term alterations and may involve compensatory mechanisms. Moreover, partial depletion of NGs as performed in the present manuscript may even be of higher clinical relevance. In our study, we did not functionally test a potential contribution of BBB integrity and signaling or the causal effect of NG migration on the brain. To expand on the present findings, future studies could investigate the impact of NG migration to the brain through inhibition of the NG recruitment receptor CXCR2, which metabolic pathways are altered by neutropenia during septic-like inflammation, and further assess humoral interactions with brain endothelial cells and CVOs. The contributions of NETs in the periphery and brain to sickness responses should also be analyzed in a more dynamic manner i.e. at different time points and brain structures. Here, we identified a significant modulatory role of NGs on peripheral inflammation and thermoregulation. The present data are meaningful and recognize the beneficial role of NGs during septic-like inflammation with strong implications for animals and patients suffering from neutropenia.

In summary, during high-dose-LPS-induced inflammation, partial NG depletion reduced NG recruitment to the brain in a structure dependent manner and exacerbated sickness responses. We confirm that partial NG depletion amplifies peripheral inflammatory responses, dysregulates the HPA-axis, and exacerbates hypothermia. Moderate differences in hypothalamic activation markers between neutropenic and immunocompetent mice (i.e. IL-6, CXCL2, and CCL5) suggest that a peripheral effect was more important than a direct effect on the brain in regulating sickness response severity, at least in the model and circumstances investigated here. In particular, elevated levels of IL-10 but potentially also IL-6 and TNFα interacting at the BBB and CVOs and in the periphery most likely contribute to severe hypothermia. These data indicate a direct or indirect anti-inflammatory role of NGs and could partially explain the high mortality documented in clinical cases of neutropenic fever even independent of bacterial growth. Though treatment strategies for patients with or without neutropenia are largely the same, tailored therapies for neutropenic patients could help improve overall outcomes in the future and should be further explored.

## Data Availability

The original contributions presented in the study are included in the article/**Supplementary Material**. Further inquiries can be directed to the corresponding author/s.

## References

[1] Semmler A Hermann S Mormann F Weberpals M Paxian SA Okulla T. Sepsis causes neuroinflammation and concomitant decrease of cerebral metabolism. J Neuroinflammation. (2008) 5:38. doi: 10.1186/1742-2094-5-38 18793399 PMC2553764

[2] Karampela I Fragkou PC. Future perspectives in the diagnosis and treatment of sepsis and septic shock. Medicina (Kaunas). (2022) 58. doi: 10.3390/medicina58070844 PMC9323821 35888563

[3] Schiel X Hebart H Kern WV Kiehl MG Solch JP Wilhelm S. Sepsis in neutropenia–guidelines of the infectious diseases working party (AGIHO) of the german society of hematology and oncology (DGHO). Ann Hematol. (2003) 82 Suppl 2:S158–66. doi: 10.1007/s00277-003-0770-6 13680167

[4] Wiersinga WJ Leopold SJ Cranendonk DR van der Poll T. Host innate immune responses to sepsis. Virulence. (2014) 5:36–44. doi: 10.4161/viru.25436 23774844 PMC3916381

[5] Pizzo PA. Management of fever in patients with cancer and treatment-induced neutropenia. N Engl J Med. (1993) 328:1323–32. doi: 10.1056/NEJM199305063281808 8469254

[6] Gentile LF Cuenca AG Efron PA Ang D Bihorac A McKinley BA. Persistent inflammation and immunosuppression: a common syndrome and new horizon for surgical intensive care. J Trauma Acute Care Surg. (2012) 72:1491–501. doi: 10.1097/TA.0b013e318256e000 PMC3705923 22695412

[7] Biff D Petronilho F Constantino L Vuolo F Zamora-Berridi GJ Dall’Igna DM. Correlation of acute phase inflammatory and oxidative markers with long-term cognitive impairment in sepsis survivors rats. Shock. (2013) 40:45–8. doi: 10.1097/SHK.0b013e3182959cfa 23603768

[8] Correa TD Rocha LL Pessoa CM Silva E de Assuncao MS. Fluid therapy for septic shock resuscitation: which fluid should be used? Einstein (Sao Paulo). (2015) 13:462–8. doi: 10.1590/S1679-45082015RW3273 PMC4943797 26313437

[9] Lyman GH Abella E Pettengell R. Risk factors for febrile neutropenia among patients with cancer receiving chemotherapy: A systematic review. Crit Rev Oncol Hematol. (2014) 90:190–9. doi: 10.1016/j.critrevonc.2013.12.006 24434034

[10] Heinz WJ Buchheidt D Christopeit M von Lilienfeld-Toal M Cornely OA Einsele H. Diagnosis and empirical treatment of fever of unknown origin (FUO) in adult neutropenic patients: guidelines of the Infectious Diseases Working Party (AGIHO) of the German Society of Hematology and Medical Oncology (DGHO). Ann Hematol. (2017) 96:1775–92. doi: 10.1007/s00277-017-3098-3 PMC5645428 28856437

[11] Islas-Munoz B Volkow-Fernandez P Silva-Zamora J Ramirez-Ibarguen A Cornejo-Juarez P. Mortality in patients with hematological Malignancies, febrile neutropenia, and septic shock. J Infect Dev Ctries. (2024) 18:235–42. doi: 10.3855/jidc.17451 38484344

[12] Lyon JF Sadrolashrafi M Hayes MM. Febrile neutropenia. ATS Sch. (2024) 5:460–1. doi: 10.34197/ats-scholar.2023-0080OT PMC11448829 39371232

[13] Keck JM Wingler MJB Cretella DA Vijayvargiya P Wagner JL Barber KE. Approach to fever in patients with neutropenia: a review of diagnosis and management. Ther Adv Infect Dis. (2022) 9:20499361221138346. doi: 10.1177/20499361221138346 36451936 PMC9703488

[14] Delano MJ Ward PA. The immune system’s role in sepsis progression, resolution, and long-term outcome. Immunol Rev. (2016) 274:330–53. doi: 10.1111/imr.2016.274.issue-1 PMC5111634 27782333

[15] Santacroce E D’Angerio M Ciobanu AL Masini L Lo Tartaro D Coloretti I. Advances and challenges in sepsis management: modern tools and future directions. Cells. (2024) 13. doi: 10.3390/cells13050439 PMC10931424 38474403

[16] Kasten KR Muenzer JT Caldwell CC. Neutrophils are significant producers of IL-10 during sepsis. Biochem Biophys Res Commun. (2010) 393:28–31. doi: 10.1016/j.bbrc.2010.01.066 20097159 PMC2830356

[17] Brinkmann V Reichard U Goosmann C Fauler B Uhlemann Y Weiss DS. Neutrophil extracellular traps kill bacteria. Science. (2004) 303:1532–5. doi: 10.1126/science.1092385 15001782

[18] Kambas K Mitroulis I Apostolidou E Girod A Chrysanthopoulou A Pneumatikos I. Autophagy mediates the delivery of thrombogenic tissue factor to neutrophil extracellular traps in human sepsis. PloS One. (2012) 7:e45427. doi: 10.1371/journal.pone.0045427 23029002 PMC3446899

[19] Levi M van der Poll T. Coagulation and sepsis. Thromb Res. (2017) 149:38–44. doi: 10.1016/j.thromres.2016.11.007 27886531

[20] Mitroulis I Kambas K Anyfanti P Doumas M Ritis K. The multivalent activity of the tissue factor-thrombin pathway in thrombotic and non-thrombotic disorders as a target for therapeutic intervention. Expert Opin Ther Targets. (2011) 15:75–89. doi: 10.1517/14728222.2011.532788 21062231

[21] Claushuis TA van Vught LA Scicluna BP Wiewel MA Klein Klouwenberg PM Hoogendijk AJ. Thrombocytopenia is associated with a dysregulated host response in critically ill sepsis patients. Blood. (2016) 127:3062–72. doi: 10.1182/blood-2015-11-680744 26956172

[22] Schroder AK von der Ohe M Kolling U Altstaedt J Uciechowski P Fleischer D. Polymorphonuclear leucocytes selectively produce anti-inflammatory interleukin-1 receptor antagonist and chemokines, but fail to produce pro-inflammatory mediators. Immunology. (2006) 119:317–27. doi: 10.1111/j.1365-2567.2006.02435.x PMC1819575 17067311

[23] Garcia-Bonilla L Moore JM Racchumi G Zhou P Butler JM Iadecola C. Inducible nitric oxide synthase in neutrophils and endothelium contributes to ischemic brain injury in mice. J Immunol. (2014) 193:2531–7. doi: 10.4049/jimmunol.1400918 PMC4147670 25038255

[24] Hart BL. Biological basis of the behavior of sick animals. Neurosci Biobehav Rev. (1988) 12:123–37. doi: 10.1016/S0149-7634(88)80004-6 3050629

[25] Kelley KW Kent S. The legacy of sickness behaviors. Front Psychiatry. (2020) 11:607269. doi: 10.3389/fpsyt.2020.607269 33343432 PMC7744348

[26] Lasselin J. Back to the future of psychoneuroimmunology: Studying inflammation-induced sickness behavior. Brain Behav Immun Health. (2021) 18:100379. doi: 10.1016/j.bbih.2021.100379 34761246 PMC8566772

[27] Iwashyna TJ Ely EW Smith DM Langa KM. Long-term cognitive impairment and functional disability among survivors of severe sepsis. JAMA. (2010) 304:1787–94. doi: 10.1001/jama.2010.1553 PMC3345288 20978258

[28] Aguilar-Valles A Kim J Jung S Woodside B Luheshi GN. Role of brain transmigrating neutrophils in depression-like behavior during systemic infection. Mol Psychiatry. (2014) 19:599–606. doi: 10.1038/mp.2013.137 24126927

[29] Comim CM Vilela MC Constantino LS Petronilho F Vuolo F Lacerda-Queiroz N. Traffic of leukocytes and cytokine up-regulation in the central nervous system in sepsis. Intensive Care Med. (2011) 37:711–8. doi: 10.1007/s00134-011-2151-2 21350907

[30] Annane D Sharshar T. Cognitive decline after sepsis. Lancet Respir Med. (2015) 3:61–9. doi: 10.1016/S2213-2600(14)70246-2 25434614

[31] Erikson K Tuominen H Vakkala M Liisanantti JH Karttunen T Syrjala H. Brain tight junction protein expression in sepsis in an autopsy series. Crit Care. (2020) 24:385. doi: 10.1186/s13054-020-03101-3 32600371 PMC7325252

[32] Rummel C. Inflammatory transcription factors as activation markers and functional readouts in immune-to-brain communication. Brain Behav Immun. (2016) 54:1–14. doi: 10.1016/j.bbi.2015.09.003 26348582

[33] Pflieger FJ Hernandez J Schweighofer H Herden C Rosengarten B Rummel C. The role of neutrophil granulocytes in immune-to-brain communication. Temperature (Austin). (2018) 5:296–307. doi: 10.1080/23328940.2018.1538598 30574524 PMC6298491

[34] Dantzer R Konsman JP Bluthe RM Kelley KW. Neural and humoral pathways of communication from the immune system to the brain: parallel or convergent? Auton Neurosci. (2000) 85:60–5. doi: 10.1016/S1566-0702(00)00220-4 11189027

[35] Roth J Blatteis CM. Mechanisms of fever production and lysis: lessons from experimental LPS fever. Compr Physiol. (2014) 4:1563–604. doi: 10.1002/j.2040-4603.2014.tb00582.x 25428854

[36] Roth J Harre EM Rummel C Gerstberger R Hubschle T. Signaling the brain in systemic inflammation: role of sensory circumventricular organs. Front Biosci. (2004) 9:290–300. doi: 10.2741/1241 14766367

[37] Hernandez J Schaffer J Herden C Pflieger FJ Reiche S Korber S. n-3 polyunsaturated fatty acids modulate LPS-induced ARDS and the lung-brain axis of communication in wild-type versus fat-1 mice genetically modified for leukotriene B4 receptor 1 or chemerin receptor 23 knockout. Int J Mol Sci. (2023) 24. doi: 10.3390/ijms241713524 PMC10487657 37686333

[38] D’Mello C Le T Swain MG. Cerebral microglia recruit monocytes into the brain in response to tumor necrosis factoralpha signaling during peripheral organ inflammation. J Neurosci. (2009) 29:2089–102. doi: 10.1523/JNEUROSCI.3567-08.2009 PMC6666330 19228962

[39] McColl BW Rothwell NJ Allan SM. Systemic inflammatory stimulus potentiates the acute phase and CXC chemokine responses to experimental stroke and exacerbates brain damage via interleukin-1- and neutrophil-dependent mechanisms. J Neurosci. (2007) 27:4403–12. doi: 10.1523/JNEUROSCI.5376-06.2007 PMC6672305 17442825

[40] Perry VH Cunningham C Holmes C. Systemic infections and inflammation affect chronic neurodegeneration. Nat Rev Immunol. (2007) 7:161–7. doi: 10.1038/nri2015 17220915

[41] Rummel C Inoue W Poole S Luheshi GN. Leptin regulates leukocyte recruitment into the brain following systemic LPS-induced inflammation. Mol Psychiatry. (2010) 15:523–34. doi: 10.1038/mp.2009.98 19773811

[42] Bohatschek M Werner A Raivich G. Systemic LPS injection leads to granulocyte influx into normal and injured brain: effects of ICAM-1 deficiency. Exp Neurol. (2001) 172:137–52. doi: 10.1006/exnr.2001.7764 11681847

[43] Zhang HY James I Chen CL Besner GE. Heparin-binding epidermal growth factor-like growth factor (HB-EGF) preserves gut barrier function by blocking neutrophil-endothelial cell adhesion after hemorrhagic shock and resuscitation in mice. Surgery. (2012) 151:594–605. doi: 10.1016/j.surg.2011.10.001 22153812 PMC3307915

[44] Reino DC Palange D Feketeova E Bonitz RP Xu DZ Lu Q. Activation of toll-like receptor 4 is necessary for trauma hemorrhagic shock-induced gut injury and polymorphonuclear neutrophil priming. Shock. (2012) 38:107–14. doi: 10.1097/SHK.0b013e318257123a PMC3378823 22575992

[45] Filipovich Y Agrawal V Crawford SE Fitchev P Qu X Klein J. Depletion of polymorphonuclear leukocytes has no effect on preterm delivery in a mouse model of Escherichia coli-induced labor. Am J Obstet Gynecol. (2015) 213:697 e1–10. doi: 10.1016/j.ajog.2015.07.025 PMC4631622 26215328

[46] Dhami R Gilks B Xie C Zay K Wright JL Churg A. Acute cigarette smoke-induced connective tissue breakdown is mediated by neutrophils and prevented by alpha1-antitrypsin. Am J Respir Cell Mol Biol. (2000) 22:244–52. doi: 10.1165/ajrcmb.22.2.3809 10657946

[47] Buss NA Gavins FN Cover PO Terron A Buckingham JC. Targeting the annexin 1-formyl peptide receptor 2/ALX pathway affords protection against bacterial LPS-induced pathologic changes in the murine adrenal cortex. FASEB J. (2015) 29:2930–42. doi: 10.1096/fj.14-268375 25818588

[48] Schneiders J Fuchs F Damm J Herden C Gerstberger R Soares DM. The transcription factor nuclear factor interleukin 6 mediates pro- and anti-inflammatory responses during LPS-induced systemic inflammation in mice. Brain Behav Immun. (2015) 48:147–64. doi: 10.1016/j.bbi.2015.03.008 25813145

[49] Peek V Harden LM Damm J Aslani F Leisengang S Roth J. LPS primes brain responsiveness to high mobility group box-1 protein. Pharmaceuticals (Basel). (2021) 14. doi: 10.3390/ph14060558 PMC8230749 34208101

[50] Bredehoft J Dolga AM Honrath B Wache S Mazurek S Culmsee C. SK-channel activation alters peripheral metabolic pathways in mice, but not lipopolysaccharide-induced fever or inflammation. J Inflammation Res. (2022) 15:509–31. doi: 10.2147/JIR.S338812 PMC8800008 35115803

[51] Bauer N Moritz A. Evaluation of three methods for measurement of hemoglobin and calculated hemoglobin parameters with the ADVIA 2120 and ADVIA 120 in dogs, cats, and horses. Vet Clin Pathol. (2008) 37:173–9. doi: 10.1111/j.1939-165X.2008.00039.x 18533916

[52] Motulsky HJ Brown RE. Detecting outliers when fitting data with nonlinear regression - a new method based on robust nonlinear regression and the false discovery rate. BMC Bioinf. (2006) 7:123. doi: 10.1186/1471-2105-7-123 PMC1472692 16526949

[53] Chaudhry H Zhou J Zhong Y Ali MM McGuire F Nagarkatti PS. Role of cytokines as a double-edged sword in sepsis. In Vivo. (2013) 27:669–84. PMC4378830 24292568

[54] Damm J Wiegand F Harden LM Gerstberger R Rummel C Roth J. Fever, sickness behavior, and expression of inflammatory genes in the hypothalamus after systemic and localized subcutaneous stimulation of rats with the Toll-like receptor 7 agonist imiquimod. Neuroscience. (2012) 201:166–83. doi: 10.1016/j.neuroscience.2011.11.013 22116053

[55] MaChado NLS Saper CB. Genetic identification of preoptic neurons that regulate body temperature in mice. Temperature (Austin). (2022) 9:14–22. doi: 10.1080/23328940.2021.1993734 35655663 PMC9154766

[56] Osaka T. The EP(3) and EP(4) receptor subtypes both mediate the fever-producing effects of prostaglandin E(2) in the rostral ventromedial preoptic area of the hypothalamus in rats. Neuroscience. (2022) 494:25–37. doi: 10.1016/j.neuroscience.2022.05.001 35550162

[57] Byun DJ Lee J Yu JW Hyun YM. NLRP3 exacerbate NETosis-associated neuroinflammation in an LPS-induced inflamed brain. Immune Netw. (2023) 23:e27. doi: 10.4110/in.2023.23.e27 37416934 PMC10320420

[58] Boulant JA. Role of the preoptic-anterior hypothalamus in thermoregulation and fever. Clin Infect Dis. (2000) 31 Suppl 5:S157–61. doi: 10.1086/317521 11113018

[59] McKinley MJ McAllen RM Davern P Giles ME Penschow J Sunn N. The sensory circumventricular organs of the mammalian brain. Adv Anat Embryol Cell Biol. (2003) 172:III–XII,1-122. doi: 10.1007/978-3-642-55532-9 12901335

[60] Danikas DD Karakantza M Theodorou GL Sakellaropoulos GC Gogos CA. Prognostic value of phagocytic activity of neutrophils and monocytes in sepsis. Correlation to CD64 and CD14 antigen expression. Clin Exp Immunol. (2008) 154:87–97. doi: 10.1111/j.1365-2249.2008.03737.x 18727624 PMC2561092

[61] Tang BM McLean AS Dawes IW Huang SJ Lin RC. The use of gene-expression profiling to identify candidate genes in human sepsis. Am J Respir Crit Care Med. (2007) 176:676–84. doi: 10.1164/rccm.200612-1819OC 17575094

[62] Craciun FL Schuller ER Remick DG. Early enhanced local neutrophil recruitment in peritonitis-induced sepsis improves bacterial clearance and survival. J Immunol. (2010) 185:6930–8. doi: 10.4049/jimmunol.1002300 PMC5540308 21041722

[63] Tollner B Roth J Storr B Martin D Voigt K Zeisberger E. The role of tumor necrosis factor (TNF) in the febrile and metabolic responses of rats to intraperitoneal injection of a high dose of lipopolysaccharide. Pflugers Arch. (2000) 440:925–32. doi: 10.1007/s004240000386 11041560

[64] Leon LR. Hypothermia in systemic inflammation: role of cytokines. Front Biosci. (2004) 9:1877–88. doi: 10.2741/1381 14977594

[65] Steiner AA Hunter JC Phipps SM Nucci TB Oliveira DL Roberts JL. Cyclooxygenase-1 or -2–which one mediates lipopolysaccharide-induced hypothermia? Am J Physiol Regul Integr Comp Physiol. (2009) 297:R485–94. doi: 10.1152/ajpregu.91026.2008 PMC2724239 19515980

[66] Garami A Steiner AA Romanovsky AA. Fever and hypothermia in systemic inflammation. Handb Clin Neurol. (2018) 157:565–97. doi: 10.1016/B978-0-444-64074-1.00034-3 30459026

[67] Fonseca MT Rodrigues AC Cezar LC Fujita A Soriano FG Steiner AA. Spontaneous hypothermia in human sepsis is a transient, self-limiting, and nonterminal response. J Appl Physiol (1985). (2016) 120:1394–401. doi: 10.1152/japplphysiol.00004.2016 26989218

[68] Alberca RW Gomes E Moretti EH Russo M Steiner AA. Naturally occurring hypothermia promotes survival in severe anaphylaxis. Immunol Lett. (2021) 237:27–32. doi: 10.1016/j.imlet.2021.07.002 34245741

[69] Steiner AA Fonseca MT Soriano FG. Should we assume that hypothermia is a dysfunction in sepsis? Crit Care. (2017) 21:8. doi: 10.1186/s13054-016-1584-y 28073371 PMC5225576

[70] Rudaya AY Steiner AA Robbins JR Dragic AS Romanovsky AA. Thermoregulatory responses to lipopolysaccharide in the mouse: dependence on the dose and ambient temperature. Am J Physiol Regul Integr Comp Physiol. (2005) 289:R1244–52. doi: 10.1152/ajpregu.00370.2005 16081879

[71] Wanner SP Almeida MC Shimansky YP Oliveira DL Eales JR Coimbra CC. Cold-induced thermogenesis and inflammation-associated cold-seeking behavior are represented by different dorsomedial hypothalamic sites: A three-dimensional functional topography study in conscious rats. J Neurosci. (2017) 37:6956–71. doi: 10.1523/JNEUROSCI.0100-17.2017 PMC5518423 28630253

[72] Erratum: Wanner. Cold-induced thermogenesis and inflammation-associated cold-seeking behavior are represented by different dorsomedial hypothalamic sites: A three-dimensional functional topography study in conscious rats. J Neurosci. (2018) 38:1054. doi: 10.1523/JNEUROSCI.3541-17.2017 PMC6596230 31329691

[73] Gao Y Zhu J Yin C Zhu J Zhu T Liu L. Effects of target temperature management on the outcome of septic patients with fever. BioMed Res Int. (2017) 2017:3906032. doi: 10.1155/2017/3906032 29259979 PMC5702415

[74] Rumbus Z Matics R Hegyi P Zsiboras C Szabo I Illes A. Fever is associated with reduced, hypothermia with increased mortality in septic patients: A meta-analysis of clinical trials. PloS One. (2017) 12:e0170152. doi: 10.1371/journal.pone.0170152 28081244 PMC5230786

[75] Drewry AM Fuller BM Skrupky LP Hotchkiss RS. The presence of hypothermia within 24 hours of sepsis diagnosis predicts persistent lymphopenia. Crit Care Med. (2015) 43:1165–9. doi: 10.1097/CCM.0000000000000940 PMC4700928 25793436

[76] Kushimoto S Gando S Saitoh D Mayumi T Ogura H Fujishima S. The impact of body temperature abnormalities on the disease severity and outcome in patients with severe sepsis: an analysis from a multicenter, prospective survey of severe sepsis. Crit Care. (2013) 17:R271. doi: 10.1186/cc13106 24220071 PMC4057086

[77] Laflamme N Rivest S. Toll-like receptor 4: the missing link of the cerebral innate immune response triggered by circulating gram-negative bacterial cell wall components. FASEB J. (2001) 15:155–63. doi: 10.1096/fj.00-0339com 11149903

[78] Chakravarty S Herkenham M. Toll-like receptor 4 on nonhematopoietic cells sustains CNS inflammation during endotoxemia, independent of systemic cytokines. J Neurosci. (2005) 25:1788–96. doi: 10.1523/JNEUROSCI.4268-04.2005 PMC6725921 15716415

[79] Mallard C. Innate immune regulation by toll-like receptors in the brain. ISRN Neurol. (2012) 2012:701950. doi: 10.5402/2012/701950 23097717 PMC3477747

[80] Johnson RH Kho DT SJ OC Angel CE Graham ES. The functional and inflammatory response of brain endothelial cells to Toll-Like Receptor agonists. Sci Rep. (2018) 8:10102. doi: 10.1038/s41598-018-28518-3 29973684 PMC6031625

[81] Akira S Taga T Kishimoto T. Interleukin-6 in biology and medicine. Adv Immunol. (1993) 54:1–78. doi: 10.1016/S0065-2776(08)60532-5 8379461

[82] Quan N Whiteside M Herkenham M. Time course and localization patterns of interleukin-1beta messenger RNA expression in brain and pituitary after peripheral administration of lipopolysaccharide. Neuroscience. (1998) 83:281–93. doi: 10.1016/S0306-4522(97)00350-3 9466417

[83] Wajant H Scheurich P. TNFR1-induced activation of the classical NF-kappaB pathway. FEBS J. (2011) 278:862–76. doi: 10.1111/j.1742-4658.2011.08015.x 21232017

[84] Schlegel N Leweke R Meir M Germer CT Waschke J. Role of NF-kappaB activation in LPS-induced endothelial barrier breakdown. Histochem Cell Biol. (2012) 138:627–41. doi: 10.1007/s00418-012-0983-7 22718247

[85] Norden DM Trojanowski PJ Villanueva E Navarro E Godbout JP. Sequential activation of microglia and astrocyte cytokine expression precedes increased Iba-1 or GFAP immunoreactivity following systemic immune challenge. Glia. (2016) 64:300–16. doi: 10.1002/glia.22930 PMC4707977 26470014

[86] Saito H Sherwood ER Varma TK Evers BM. Effects of aging on mortality, hypothermia, and cytokine induction in mice with endotoxemia or sepsis. Mech Ageing Dev. (2003) 124:1047–58. doi: 10.1016/j.mad.2003.08.002 14659593

[87] Corrigan JJ Fonseca MT Flatow EA Lewis K Steiner AA. Hypometabolism and hypothermia in the rat model of endotoxic shock: independence of circulatory hypoxia. J Physiol. (2014) 592:3901–16. doi: 10.1113/tjp.2014.592.issue-17 PMC4192710 24951620

[88] Mul Fedele ML Aiello I Caldart CS Golombek DA Marpegan L Paladino N. Differential thermoregulatory and inflammatory patterns in the circadian response to LPS-induced septic shock. Front Cell Infect Microbiol. (2020) 10:100. doi: 10.3389/fcimb.2020.00100 32226779 PMC7080817

[89] Tsao N Hsu HP Wu CM Liu CC Lei HY. Tumour necrosis factor-alpha causes an increase in blood-brain barrier permeability during sepsis. J Med Microbiol. (2001) 50:812–21. doi: 10.1099/0022-1317-50-9-812 11549183

[90] Mina F Comim CM Dominguini D Cassol OJ Jr. Dall Igna DM Ferreira GK. Il1-beta involvement in cognitive impairment after sepsis. Mol Neurobiol. (2014) 49:1069–76. doi: 10.1007/s12035-013-8581-9 24234155

[91] Hu W Pasare C. Location, location, location: tissue-specific regulation of immune responses. J Leukoc Biol. (2013) 94:409–21. doi: 10.1189/jlb.0413207 PMC3747123 23825388

[92] Oda S Hirasawa H Shiga H Nakanishi K Matsuda K Nakamua M. Sequential measurement of IL-6 blood levels in patients with systemic inflammatory response syndrome (SIRS)/sepsis. Cytokine. (2005) 29:169–75. doi: 10.1016/j.cyto.2004.10.010 15652449

[93] Song J Park DW Moon S Cho HJ Park JH Seok H. Diagnostic and prognostic value of interleukin-6, pentraxin 3, and procalcitonin levels among sepsis and septic shock patients: a prospective controlled study according to the Sepsis-3 definitions. BMC Infect Dis. (2019) 19:968. doi: 10.1186/s12879-019-4618-7 31718563 PMC6852730

[94] Baghel K Srivastava RN Chandra A Goel SK Agrawal J Kazmi HR. TNF-alpha, IL-6, and IL-8 cytokines and their association with TNF-alpha-308 G/A polymorphism and postoperative sepsis. J Gastrointest Surg. (2014) 18:1486–94. doi: 10.1007/s11605-014-2574-5 24944154

[95] Quinto BM Iizuka IJ Monte JC Santos BF Pereira V Durao MS. TNF-alpha depuration is a predictor of mortality in critically ill patients under continuous veno-venous hemodiafiltration treatment. Cytokine. (2015) 71:255–60. doi: 10.1016/j.cyto.2014.10.024 25461406

[96] Reilly JP Anderson BJ Hudock KM Dunn TG Kazi A Tommasini A. Neutropenic sepsis is associated with distinct clinical and biological characteristics: a cohort study of severe sepsis. Crit Care. (2016) 20:222. doi: 10.1186/s13054-016-1398-y 27431667 PMC4950810

[97] Gharamti AA Samara O Monzon A Montalbano G Scherger S DeSanto K. Proinflammatory cytokines levels in sepsis and healthy volunteers, and tumor necrosis factor-alpha associated sepsis mortality: A systematic review and meta-analysis. Cytokine. (2022) 158:156006. doi: 10.1016/j.cyto.2022.156006 36044827

[98] Cheng X Yang YL Yang H Wang YH Du GH. Kaempferol alleviates LPS-induced neuroinflammation and BBB dysfunction in mice via inhibiting HMGB1 release and down-regulating TLR4/MyD88 pathway. Int Immunopharmacol. (2018) 56:29–35. doi: 10.1016/j.intimp.2018.01.002 29328946

[99] Voirin AC Perek N Roche F. Inflammatory stress induced by a combination of cytokines (IL-6, IL-17, TNF-alpha) leads to a loss of integrity on bEnd.3 endothelial cells *in vitro* BBB model. Brain Res. (2020) 1730:146647. doi: 10.1016/j.brainres.2020.146647 31911168

[100] Alexander JJ Jacob A Cunningham P Hensley L Quigg RJ. TNF is a key mediator of septic encephalopathy acting through its receptor, TNF receptor-1. Neurochem Int. (2008) 52:447–56. doi: 10.1016/j.neuint.2007.08.006 PMC3191465 17884256

[101] Leon LR White AA Kluger MJ. Role of IL-6 and TNF in thermoregulation and survival during sepsis in mice. Am J Physiol. (1998) 275:R269–77. doi: 10.1152/ajpregu.1998.275.1.R269 9688988

[102] Remick DG Bolgos G Copeland S Siddiqui J. Role of interleukin-6 in mortality from and physiologic response to sepsis. Infect Immun. (2005) 73:2751–7. doi: 10.1128/IAI.73.5.2751-2757.2005 PMC1087378 15845478

[103] Wu HP Chen CK Chung K Tseng JC Hua CC Liu YC. Serial cytokine levels in patients with severe sepsis. Inflammation Res. (2009) 58:385–93. doi: 10.1007/s00011-009-0003-0 19262987

[104] Yin F Xi YL Wang Y Li BR Qian J Ren H. The clinical outcomes and biomarker features of severe sepsis/septic shock with severe neutropenia: a retrospective cohort study. Transl Pediatr. (2021) 10:464–73. doi: 10.21037/tp-20-230 PMC8039791 33850805

[105] Fabri A Kandara K Coudereau R Gossez M Abraham P Monard C. Characterization of circulating IL-10-producing cells in septic shock patients: A proof of concept study. Front Immunol. (2020) 11:615009. doi: 10.3389/fimmu.2020.615009 33613540 PMC7890231

[106] Abe R Hirasawa H Oda S Sadahiro T Nakamura M Watanabe E. Up-regulation of interleukin-10 mRNA expression in peripheral leukocytes predicts poor outcome and diminished human leukocyte antigen-DR expression on monocytes in septic patients. J Surg Res. (2008) 147:1–8. doi: 10.1016/j.jss.2007.07.009 17720196

[107] Li X Xu Z Pang X Huang Y Yang B Yang Y. Interleukin-10/lymphocyte ratio predicts mortality in severe septic patients. PloS One. (2017) 12:e0179050. doi: 10.1371/journal.pone.0179050 28628675 PMC5476240

[108] van Vught LA Wiewel MA Hoogendijk AJ Frencken JF Scicluna BP Klein Klouwenberg PMC. The host response in patients with sepsis developing intensive care unit-acquired secondary infections. Am J Respir Crit Care Med. (2017) 196:458–70. doi: 10.1164/rccm.201606-1225OC 28107024

[109] Harden LM Rummel C Laburn HP Damm J Wiegand F Poole S. Critical role for peripherally-derived interleukin-10 in mediating the thermoregulatory manifestations of fever and hypothermia in severe forms of lipopolysaccharide-induced inflammation. Pflugers Arch. (2014) 466:1451–66. doi: 10.1007/s00424-013-1371-4 24114176

[110] Al-Saffar H Lewis K Liu E Schober A Corrigan JJ Shibata K. Lipopolysaccharide-induced hypothermia and hypotension are associated with inflammatory signaling that is triggered outside the brain. Brain Behav Immun. (2013) 28:188–95. doi: 10.1016/j.bbi.2012.11.015 23207106

[111] Chrousos GP. The hypothalamic-pituitary-adrenal axis and immune-mediated inflammation. N Engl J Med. (1995) 332:1351–62. doi: 10.1056/NEJM199505183322008 7715646

[112] Fuchs F Damm J Gerstberger R Roth J Rummel C. Activation of the inflammatory transcription factor nuclear factor interleukin-6 during inflammatory and psychological stress in the brain. J Neuroinflammation. (2013) 10:140. doi: 10.1186/1742-2094-10-140 24279606 PMC4222273

[113] Damm J Luheshi GN Gerstberger R Roth J Rummel C. Spatiotemporal nuclear factor interleukin-6 expression in the rat brain during lipopolysaccharide-induced fever is linked to sustained hypothalamic inflammatory target gene induction. J Comp Neurol. (2011) 519:480–505. doi: 10.1002/cne.v519.3 21192080

[114] Boonen E Vervenne H Meersseman P Andrew R Mortier L Declercq PE. Reduced cortisol metabolism during critical illness. N Engl J Med. (2013) 368:1477–88. doi: 10.1056/NEJMoa1214969 PMC4413428 23506003

[115] Silverman MN Pearce BD Biron CA Miller AH. Immune modulation of the hypothalamic-pituitary-adrenal (HPA) axis during viral infection. Viral Immunol. (2005) 18:41–78. doi: 10.1089/vim.2005.18.41 15802953 PMC1224723

[116] Mehet DK Philip J Solito E Buckingham JC John CD. Evidence from *in vitro* and *in vivo* studies showing that nuclear factor-kappaB within the pituitary folliculostellate cells and corticotrophs regulates adrenocorticotrophic hormone secretion in experimental endotoxaemia. J Neuroendocrinol. (2012) 24:862–73. doi: 10.1111/j.1365-2826.2012.02285.x 22283629

[117] Bollaert PE Fieux F Charpentier C Levy B. Baseline cortisol levels, cortisol response to corticotropin, and prognosis in late septic shock. Shock. (2003) 19:13–5. doi: 10.1097/00024382-200301000-00003 12558137

[118] Carlson DE Chiu WC. The absence of circadian cues during recovery from sepsis modifies pituitary-adrenocortical function and impairs survival. Shock. (2008) 29:127–32. doi: 10.1097/shk.0b013e318142c5a2 17693947

[119] Wang H Hong LJ Huang JY Jiang Q Tao RR Tan C. P2RX7 sensitizes Mac-1/ICAM-1-dependent leukocyte-endothelial adhesion and promotes neurovascular injury during septic encephalopathy. Cell Res. (2015) 25:674–90. doi: 10.1038/cr.2015.61 PMC4456628 25998681

[120] Papayannopoulos V Metzler KD Hakkim A Zychlinsky A. Neutrophil elastase and myeloperoxidase regulate the formation of neutrophil extracellular traps. J Cell Biol. (2010) 191:677–91. doi: 10.1083/jcb.201006052 PMC3003309 20974816

[121] Singh RK Liao W Tracey-White D Recchi C Tolmachova T Rankin SM. Rab27a-mediated protease release regulates neutrophil recruitment by allowing uropod detachment. J Cell Sci. (2012) 125:1652–6. doi: 10.1242/jcs.100438 PMC3346826 22375060

[122] Makrygianni EA Chrousos GP. Neural progenitor cells and the hypothalamus. Cells. (2023) 12. doi: 10.3390/cells12141822 PMC10378393 37508487

[123] Yan X Yang K Xiao Q Hou R Pan X Zhu X. Central role of microglia in sepsis-associated encephalopathy: From mechanism to therapy. Front Immunol. (2022) 13:929316. doi: 10.3389/fimmu.2022.929316 35958583 PMC9361477

[124] Shih RH Wang CY Yang CM. NF-kappaB signaling pathways in neurological inflammation: A mini review. Front Mol Neurosci. (2015) 8:77. doi: 10.3389/fnmol.2015.00077 26733801 PMC4683208

[125] Damm J Harden LM Gerstberger R Roth J Rummel C. The putative JAK-STAT inhibitor AG490 exacerbates LPS-fever, reduces sickness behavior, and alters the expression of pro- and anti-inflammatory genes in the rat brain. Neuropharmacology. (2013) 71:98–111. doi: 10.1016/j.neuropharm.2013.03.014 23548702

[126] Wung BS Ni CW Wang DL. ICAM-1 induction by TNFalpha and IL-6 is mediated by distinct pathways via Rac in endothelial cells. J BioMed Sci. (2005) 12:91–101. doi: 10.1007/s11373-004-8170-z 15864742

[127] Manzel LJ Chin CL Behlke MA Look DC. Regulation of bacteria-induced intercellular adhesion molecule-1 by CCAAT/enhancer binding proteins. Am J Respir Cell Mol Biol. (2009) 40:200–10. doi: 10.1165/rcmb.2008-0104OC PMC2633142 18703796

[128] Rummel C Voss T Matsumura K Korte S Gerstberger R Roth J. Nuclear STAT3 translocation in Guinea pig and rat brain endothelium during systemic challenge with lipopolysaccharide and interleukin-6. J Comp Neurol. (2005) 491:1–14. doi: 10.1002/cne.v491:1 16127698

[129] Rummel C Sachot C Poole S Luheshi GN. Circulating interleukin-6 induces fever through a STAT3-linked activation of COX-2 in the brain. Am J Physiol Regul Integr Comp Physiol. (2006) 291:R1316–26. doi: 10.1152/ajpregu.00301.2006 16809483

[130] Beurel E Jope RS. Lipopolysaccharide-induced interleukin-6 production is controlled by glycogen synthase kinase-3 and STAT3 in the brain. J Neuroinflammation. (2009) 6:9. doi: 10.1186/1742-2094-6-9 19284588 PMC2660311

[131] Datta SC Opp MR. Lipopolysaccharide-induced increases in cytokines in discrete mouse brain regions are detectable using Luminex xMAP technology. J Neurosci Methods. (2008) 175:119–24. doi: 10.1016/j.jneumeth.2008.08.007 PMC2596194 18771691

[132] Chai Z Gatti S Toniatti C Poli V Bartfai T. Interleukin (IL)-6 gene expression in the central nervous system is necessary for fever response to lipopolysaccharide or IL-1 beta: a study on IL-6-deficient mice. J Exp Med. (1996) 183:311–6. doi: 10.1084/jem.183.1.311 PMC2192408 8551238

[133] Eskilsson A Mirrasekhian E Dufour S Schwaninger M Engblom D Blomqvist A. Immune-induced fever is mediated by IL-6 receptors on brain endothelial cells coupled to STAT3-dependent induction of brain endothelial prostaglandin synthesis. J Neurosci. (2014) 34:15957–61. doi: 10.1523/JNEUROSCI.3520-14.2014 PMC6608482 25429137

[134] Wilhelms DB Kirilov M Mirrasekhian E Eskilsson A Kugelberg UO Klar C. Deletion of prostaglandin E2 synthesizing enzymes in brain endothelial cells attenuates inflammatory fever. J Neurosci. (2014) 34:11684–90. doi: 10.1523/JNEUROSCI.1838-14.2014 PMC6608410 25164664

[135] Akanuma S Uchida Y Ohtsuki S Tachikawa M Terasaki T Hosoya K. Attenuation of prostaglandin E2 elimination across the mouse blood-brain barrier in lipopolysaccharide-induced inflammation and additive inhibitory effect of cefmetazole. Fluids Barriers CNS. (2011) 8:24. doi: 10.1186/2045-8118-8-24 22014165 PMC3224590

[136] Dalvi S Nguyen HH On N Mitchell RW Aukema HM Miller DW. Exogenous arachidonic acid mediates permeability of human brain microvessel endothelial cells through prostaglandin E2 activation of EP3 and EP4 receptors. J Neurochem. (2015) 135:867–79. doi: 10.1111/jnc.2015.135.issue-5 25865705

[137] Serrats J Schiltz JC Garcia-Bueno B van Rooijen N Reyes TM Sawchenko PE. Dual roles for perivascular macrophages in immune-to-brain signaling. Neuron. (2010) 65:94–106. doi: 10.1016/j.neuron.2009.11.032 20152116 PMC2873837

[138] Serrats J Grigoleit JS Alvarez-Salas E Sawchenko PE. Pro-inflammatory immune-to-brain signaling is involved in neuroendocrine responses to acute emotional stress. Brain Behav Immun. (2017) 62:53–63. doi: 10.1016/j.bbi.2017.02.003 28179107

[139] Minano FJ Tavares E Maldonado R. Role of endogenous macrophage inflammatory protein-2 in regulating fever induced by bacterial endotoxin in normal and immunosuppressed rats. Clin Exp Pharmacol Physiol. (2004) 31:723–31. doi: 10.1111/j.1440-1681.2004.04086.x 15554915

[140] Kong Y He G Zhang X Li J. The role of neutrophil extracellular traps in lipopolysaccharide-induced depression-like behaviors in mice. Brain Sci. (2021) 11. doi: 10.3390/brainsci11111514 PMC8615738 34827513

[141] Zhu CL Xie J Liu Q Wang Y Li HR Yu CM. PD-L1 promotes GSDMD-mediated NET release by maintaining the transcriptional activity of Stat3 in sepsis-associated encephalopathy. Int J Biol Sci. (2023) 19:1413–29. doi: 10.7150/ijbs.79913 PMC10086742 37056920

[142] Hernandez J. A role for neutrophil granulocytes as afferent signals in immune-to-brain communication during systemic and localized organ-specific lung inflammation. Giessen, Germany: Justus Liebig University Giessen (2024).

