# Supplementary material for: Partial depletion of circulating neutrophil granulocytes in mice exacerbates the inflammatory response and hypothermia during LPS induced severe systemic inflammation
Source: Front Immunol. 2025 Jun 4;16:1578590. doi: 10.3389/fimmu.2025.1578590 (PMC12173888; doi:10.3389/fimmu.2025.1578590)
Supplement: Supplementary file 1 [file DataSheet1.pdf]

Supplementary Material

Supplementary Table 1. Outliers

Figure	Time point (h)	Tissue	Analyte	NRS+ PBS	PMN+ PBS	NRS+ LPS	PMN+ LPS
2	4	serum	TNFα	1	2		
2	4	serum	IL-6		1		
2	4	serum	IL-10	1	1	1	
2	4	serum	CCL5	1			
2	24	serum	IL-6	4	4	3	1
2	24	serum	IL-10	3	2		
2	24	serum	CXCL1			1	
2	24	serum	CXCL2		1		
2	24	serum	CCL5	2			
3	24	SFO	neutrophils		1		1
3	24	PVN	neutrophils		1		
4	4	hypothalamus	IL-6	1*			
4	4	hypothalamus	IL-10	2*	2*		
4	4	hypothalamus	SOCS3	1			
4	4	hypothalamus	NF-IL6	1			
4	24	hypothalamus	IL-6	2		1*	
4	24	hypothalamus	TNFα	2			
4	24	hypothalamus	IL-10	1, 2*	2*	3*	
4	24	hypothalamus	SOCS3	2			
4	24	hypothalamus	NFκBiα	1			
4	24	hypothalamus	NF-IL6		1		
5	24	hypothalamus	COX2	1			
5	24	hypothalamus	mPGES	1			
6	4	hypothalamus	CCL5		1		
6	4	hypothalamus	CXCL2				1*
6	4	hypothalamus	CXCL5				1*
6	24	hypothalamus	CXCL1				1
6	24	hypothalamus	CXCL2	1	2*		
SF. 3	24	bloodsmear	lymphocytes				1
SF. 6	4	hypothalamus	CXCL1	2*			
SF. 6	4	hypothalamus	CD68	1		1	
SF. 6	24	hypothalamus	CXCL1	2			1
SF. 6	24	hypothalamus	Elane	1	1		
SF. 6	24	hypothalamus	CD68		1		1
*undetected							

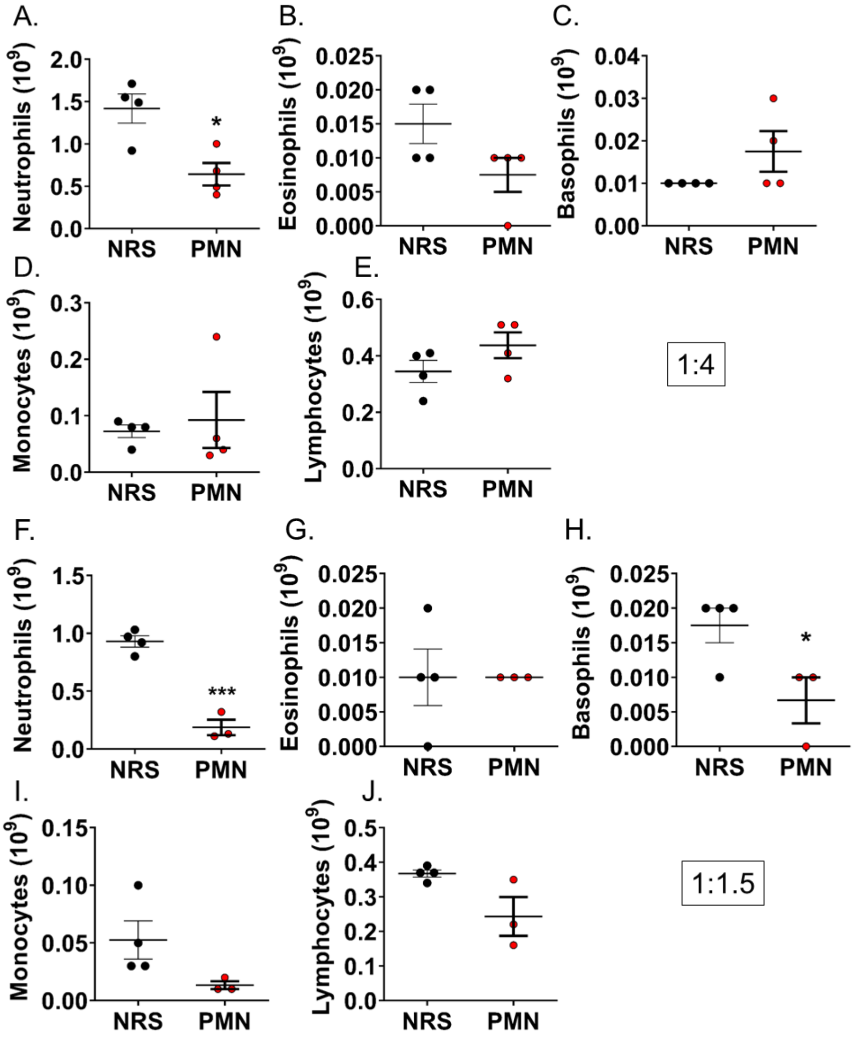

Supplementary Figure 1. Preliminary experiments to assess plasma leukocyte populations revealed that neutrophil granulocytes (neutrophils) were reduced in circulation by pre-treatment with anti-polymorphonuclear serum (PMN) in a dose dependent manner. **(A-J)** Hematological measurements with the ADVIA 2120 automated hematology analyzer of leukocyte counts in blood 24 h after IP injection at the indicated dilution ratios of PMN (A-E, 1:4 versus F-J, 1:1.5) compared to normal rabbit serum (NRS). Please note, while neither eosinophil granulocytes (eosinophils), monocytes or lymphocytes were significantly altered by PMN versus NRS treatment, the higher concentration of PMN significantly reduced circulating basophil granulocyte (basolphils) counts in addition to neutrophil granulocytes. Data are presented as dotplots with mean ± SEM (n=4). Statistical analysis were performed by unpaired t-test. (* $p < 0.05$, *** $p < 0.001$).

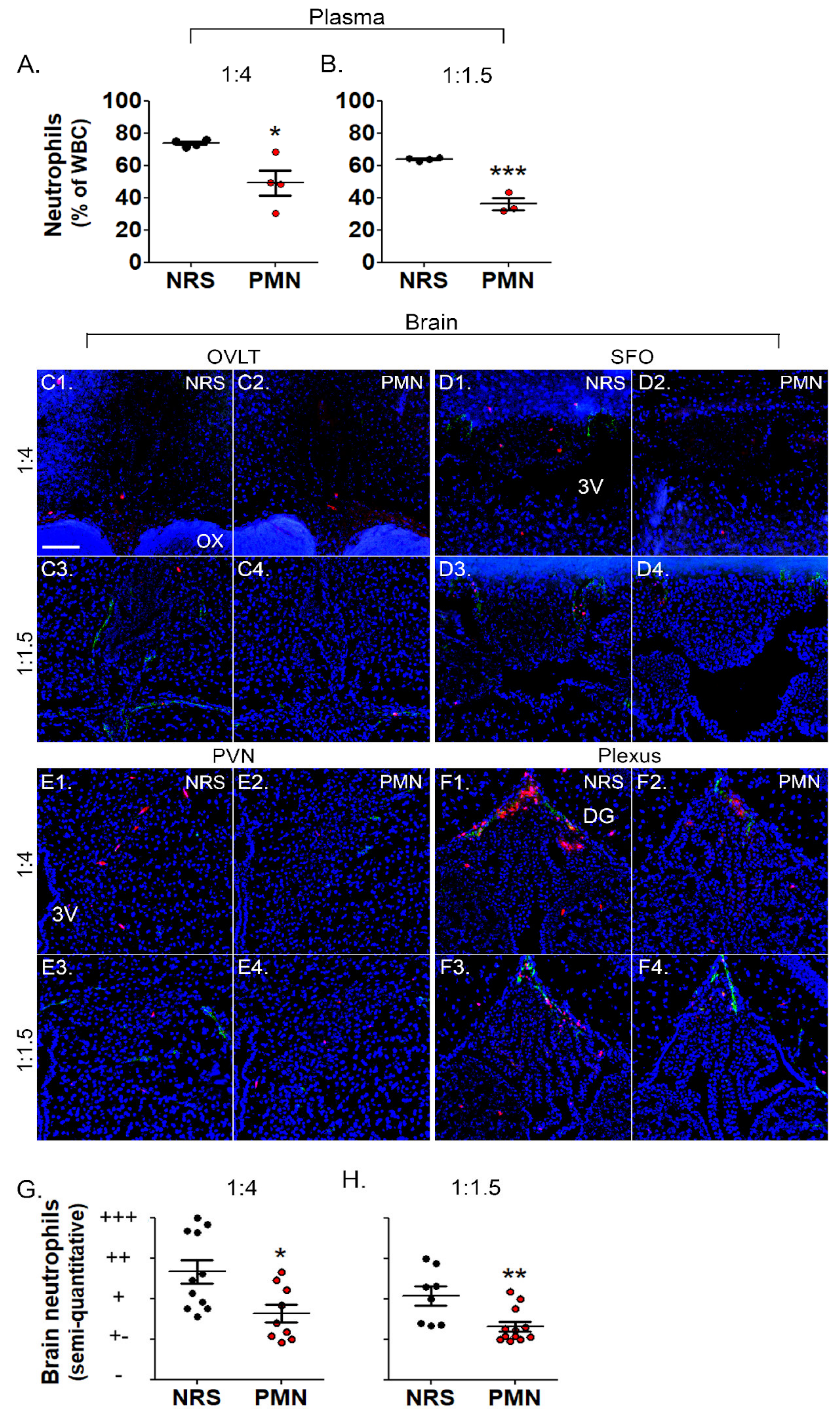

Supplementary Figure 2. Neutrophil granulocytes were reduced in circulation and in the brain during LPS-induced inflammation by pre-treatment with anti-polymorphonuclear serum (PMN) in a dose dependent manner. **(A, B)** Hematological measurements of neutrophil granulocytes presented in supplementary Fig. 1 shown as a percentage of circulating leukocytes (WBC) in plasma 24 h p.i. with LPS IP injection at the indicated dilution ratios of PMN compared to normal rabbit serum (NRS). Data are presented as dotplots with mean ± SEM (n=3-4). Statistical analysis were performed by unpaired t-test. **(C-F)**

Immunofluorescence staining of neutrophil granulocytes (red, myeloperoxidase: MPO) at the level of the vascular organ of the lamina terminalis (OVLT), subfornical organ (SFO), paraventricular nucleus (PVN), or choroid plexus (Plexus) 24 h p.i. with LPS IP injection at the indicated dilutions of PMN and NRS (scale bar = 100 µm). Dapi (blue) visualizes the surrounding tissue and von Willebrand factor (green) depicts brain vasculature. The optic nerve (OX), third ventricle (3V), and dentate gyrus (DG) are shown as structural reference points. **(G, H)** Semi-quantitative evaluation of brain neutrophil granulocytes overall, regardless of structure, were compared between PMN or NRS pre-treated mice 24 h p.i. with LPS IP injection 4 h or 24 h after stimulation. We applied a five-point scale to rate the data: +++ high, ++ moderate, + low amount of MPO staining, ± single MPO staining events, - no MPO staining. Data are presented as dotplots with mean ± SEM (n=8-12). Statistical analysis were performed by unpaired t-test. (* $p < 0.05$, ** $p < 0.01$, *** $p < 0.001$).

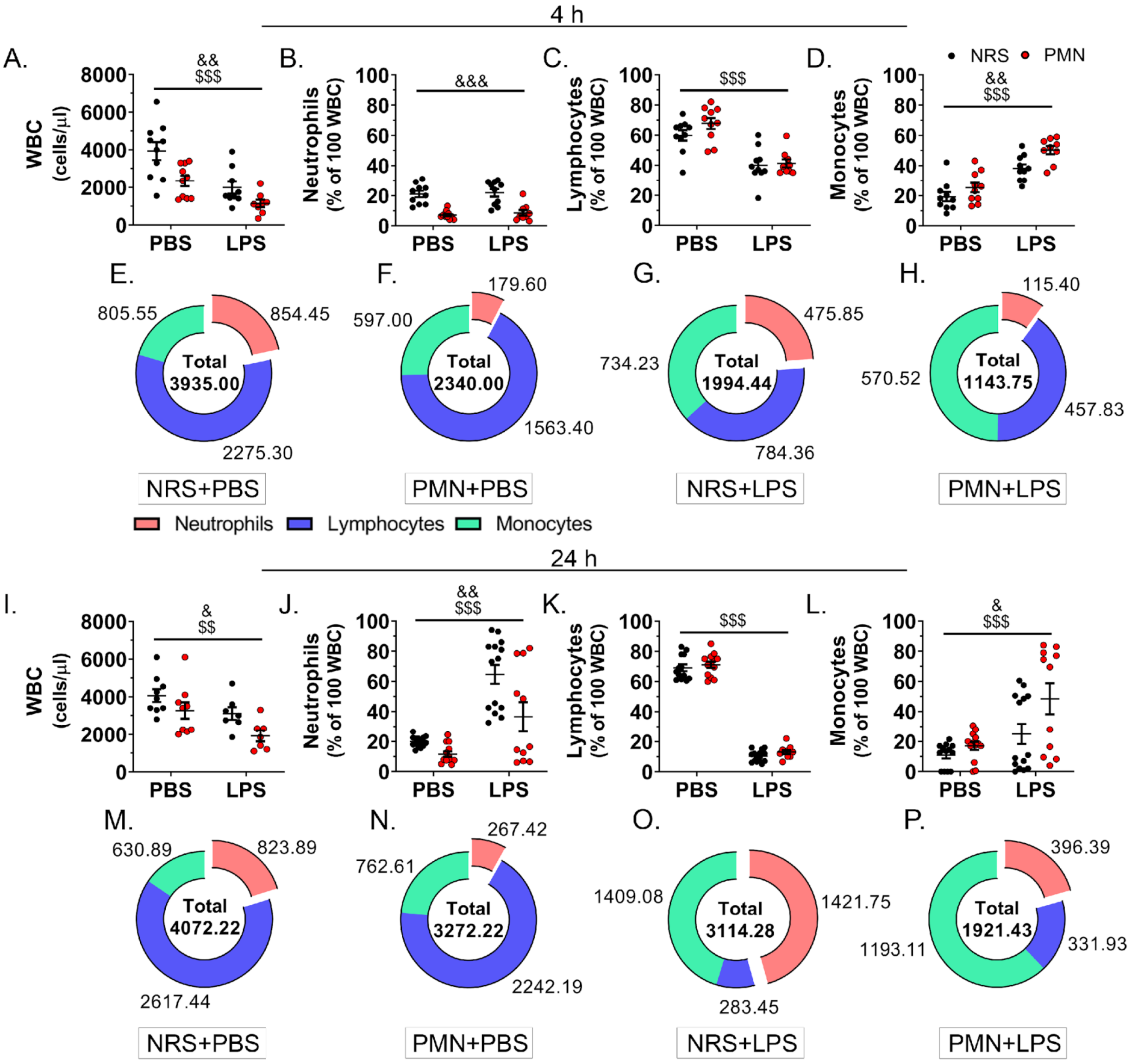

Supplementary Figure 3. Pre-treatment with PMN had little effect on lymphocyte or monocyte populations. Cellular populations in blood smears were compared between PMN or NRS pre-treated mice after PBS or LPS IP injection at the indicated time point. **(A, I)** Total WBCs. Data are presented as dotplots with mean ± SEM (n=7-10). **(B, J)** Neutrophil granulocytes. **(C, K)** Lymphocytes. **(D, L)** Monocytes. Data are presented as dotplots with mean ± SEM (n=9-14). The average raw cell counts were also shown for each treatment group at the indicated time point for neutrophil granulocytes (red), lymphocytes (blue), and monocytes (green). **(E, M)** NRS+PBS. **(F, N)** PMN+PBS. **(G, O)** NRS+LPS. **(H, P)** PMN+LPS. Statistical analysis were performed by Two-way ANOVA with the main effect: $^{\&}$PMN, $^{\$}$LPS ($^{\&}$ p< 0.05, $^{\&\&}$ p< 0.01, $^{\&\&\&}$ p< 0.001, $^{\$\$}$ p< 0.01, $^{\$\$\$}$ p< 0.001).

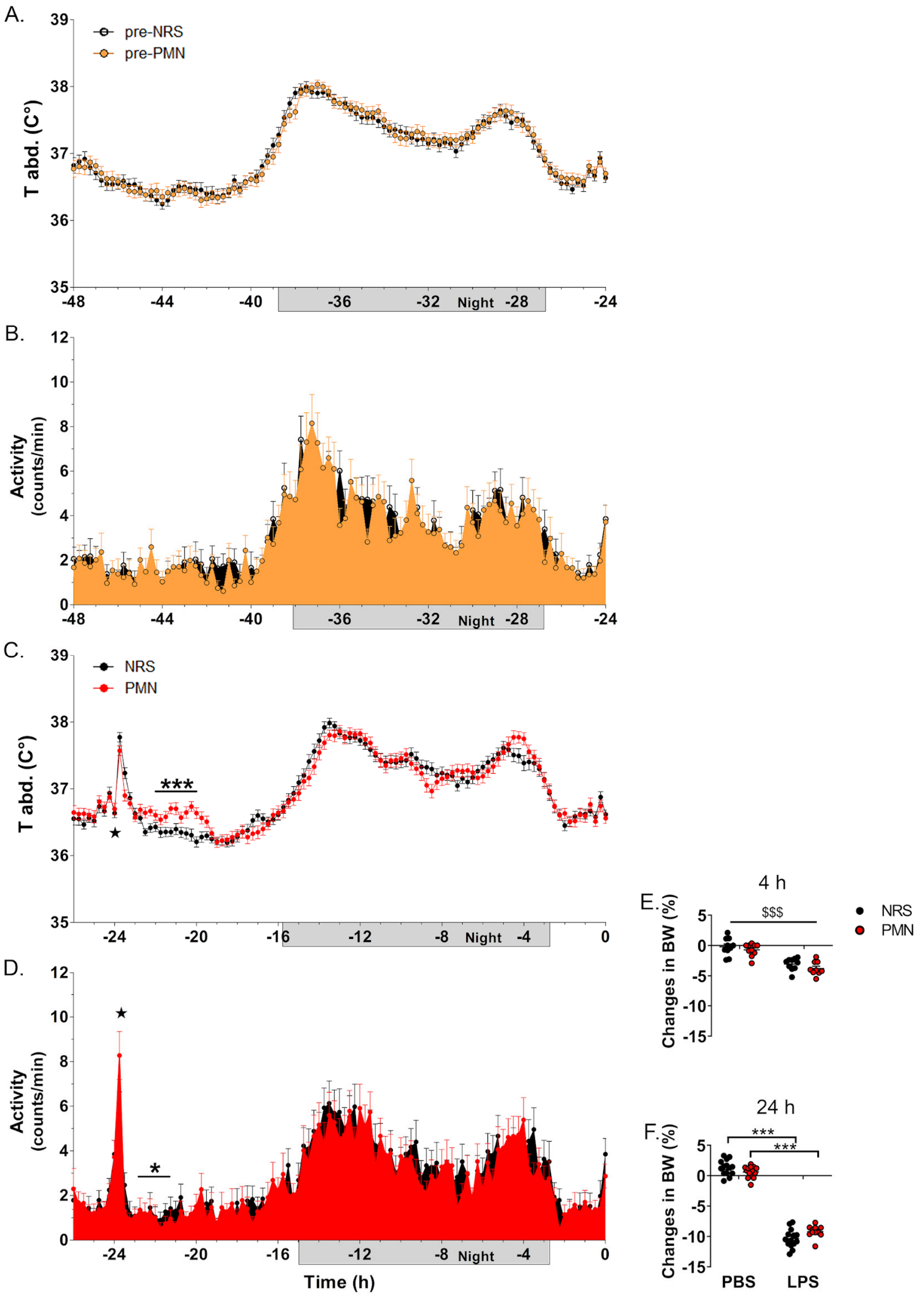

Supplementary Figure 4. Pre-treatment with PMN did not have a lasting effect on physiological parameters prior to treatment with LPS. An intra-abdominal transmitter continuously recorded the physiological parameters of mice beginning 24 h prior to pre-treatment with PMN or NRS by IP injection as indicated by

a star. **(A)** Baseline core body temperature (T abd.). **(B)** Baseline activity counts per minute. **(C)** T abd. following PMN or NRS IP injection. **(D)** Activity counts per minute following PMN or NRS IP injection. Data are presented as line graphs over time, grey boxes indicate the dark or night cycle, with mean ± SEM (n=41-48). **(E, F)** Changes in body weight (BW) were compared between PMN or NRS pre-treated mice after PBS or LPS IP injection at the indicated time point. Data are presented as dotplots with mean ± SEM (n=9-14). **(A-D)** Statistical analysis were performed by Two-way repeated measures ANOVA with effects of *PMN. **(E, F)** Statistical analysis were performed by Two-way ANOVA and Tukey post-hoc test with the main effect: $^{\$}$LPS (* $p< 0.05$, *** $p< 0.001$, $^{\$\$\$}$ $p< 0.001$).

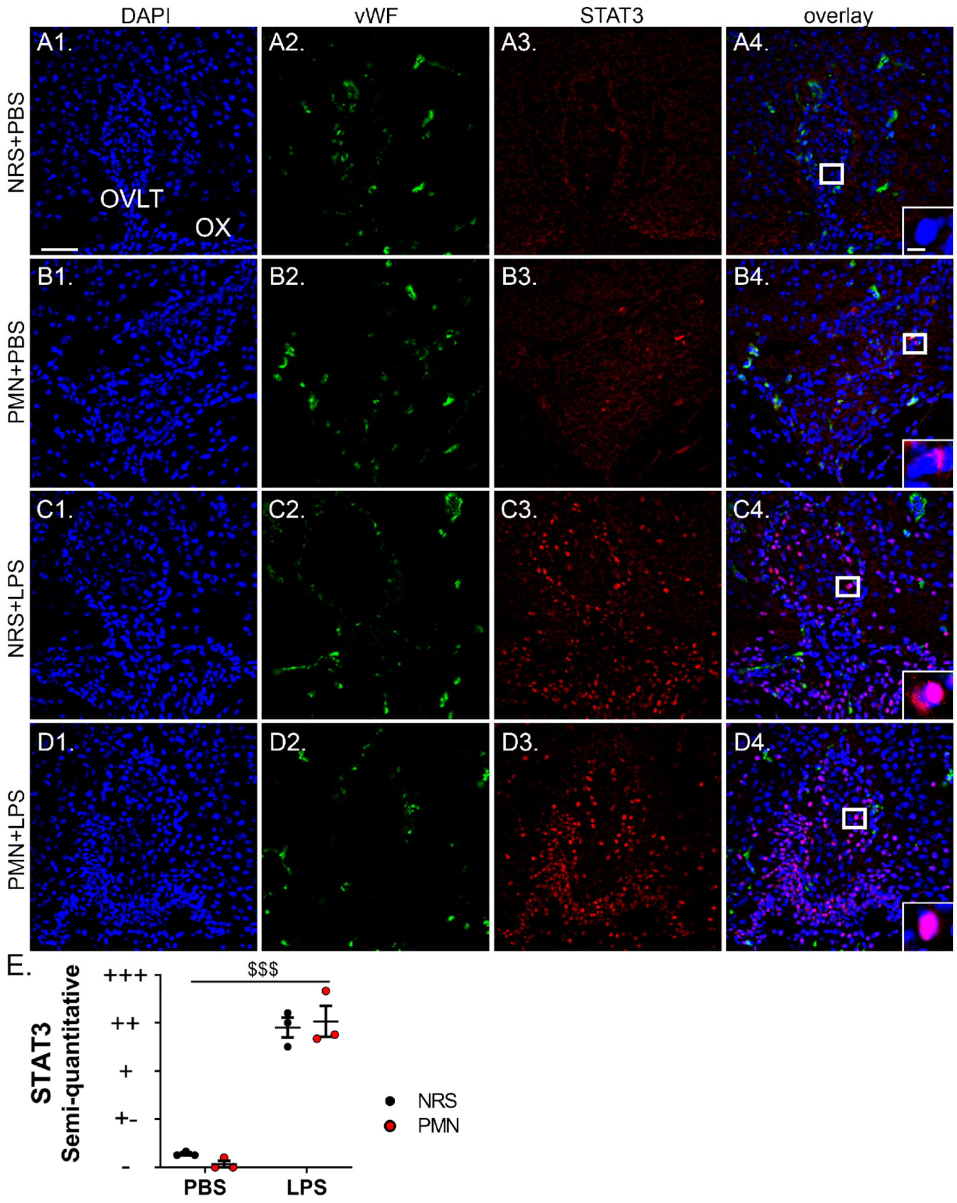

Supplementary Figure 5. LPS-induced STAT3 immunoreactivity in the brain was not altered by neutrophil granulocyte depletion. **(A-D)** Immunofluorescence staining of signal transducer and activator of transcription (STAT)3 (red) at the level of the OVLT 4 h p.i. with LPS IP injection (overview scale bar = 50 µm, insert scale bar = 5 µm). The optic nerve (OX) is shown as a structural reference point. Dapi (blue) visualizes the surrounding tissue and von Willebrand factor (vWF, green) depicts brain vasculature. We applied a five-point scale to rate the data: +++ high, ++ moderate, + low amount of nuclear translocation of STAT3, ± single nuclear STAT3 staining, - no nuclear STAT3 staining. **(E)** Semi-quantitative evaluation of nuclear STAT3 in the OVLT was compared between PMN or NRS pre-treated mice 4 h p.i. with LPS or

PBS IP injections. Data are presented as dotplots with mean ± SEM (n=3). Statistical analysis were performed by Two-way ANOVA with the main effect: $^{\$}$LPS ($^{\$\$\$}p < 0.001$).

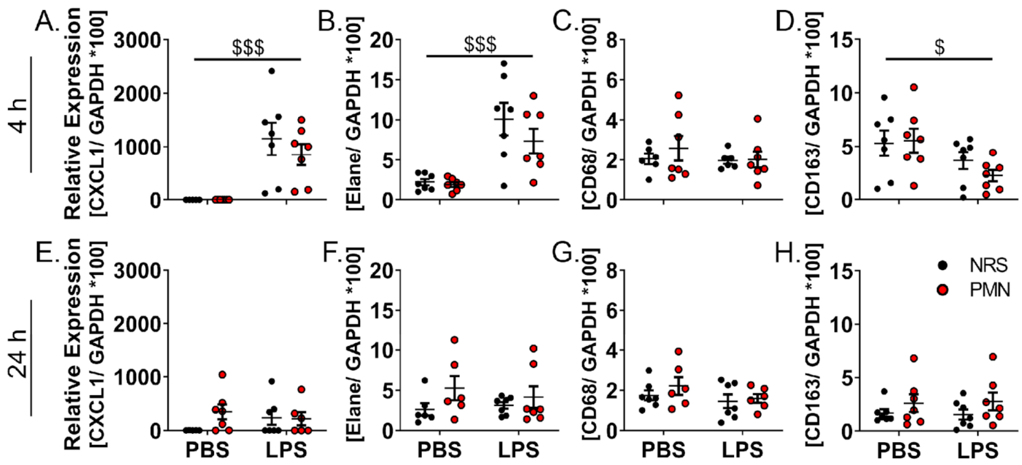

Supplementary Figure 6. Expression of neutrophil granulocyte and perivascular macrophage markers were not altered by neutrophil granulocyte depletion after PBS- or LPS-stimulation. RT-qPCR analysis of hypothalamic markers were compared between PMN or NRS pre-treated mice after PBS or LPS IP injection 4 h or 24 h after stimulation. **(A, E)** the chemokine CXCL1. **(B, F)** neutrophil elastase (ELANE). **(C, G)** the microglial marker protein CD68. **(D, H)** the marker protein for perivascular macrophages CD163. Data are presented as dotplots with mean ± SEM (n=5-7). Statistical analysis were performed by Two-way ANOVA with the main effect: $^{\$}$LPS ($^{\$}p < 0.05$, $^{\$\$\$}p < 0.001$).
